# Types of Anxiety and Depression: Theoretical Assumptions and Development of the Anxiety and Depression Questionnaire

**DOI:** 10.3389/fpsyg.2017.02376

**Published:** 2018-01-23

**Authors:** Małgorzata Fajkowska, Ewa Domaradzka, Agata Wytykowska

**Affiliations:** ^1^Institute of Psychology, Polish Academy of Sciences, Warsaw, Poland; ^2^SWPS University of Social Sciences and Humanities, Warsaw, Poland

**Keywords:** anxiety types, depression types, anxiety and depression questionnaire, measurement, reliability, validity, personality types

## Abstract

The present paper is addressed to (1) the validation of a recently proposed typology of anxiety and depression, and (2) the presentation of a new tool—the Anxiety and Depression Questionnaire (ADQ)—based on this typology. Empirical data collected across two stages—construction and validation—allowed us to offer the final form of the ADQ, designed to measure arousal anxiety, apprehension anxiety, valence depression, anhedonic depression, and mixed types of anxiety and depression. The results support the proposed typology of anxiety and depression and provide evidence that the ADQ is a reliable and valid self-rating measure of affective types, and accordingly its use in scientific research is recommended.

## Introduction: anxiety and depression as personality types

This paper is aimed at presenting the validity of a newly proposed typology of anxiety and depression, formulated within the systemic approach to personality (Fajkowska, 2013, 2015) which employed General System Theory (e.g., von Bertalanffy, 1968) and the self-report instrument that grew within this theory. The article is divided into three sections. In the Introduction section, the theoretical background of this instrument is demonstrated. In the empirical part of the paper, we report the results of our development of the Anxiety and Depression Questionnaire (ADQ) across construction (Study 1) and validation (Study 2) stages. Finally, in the Discussion section we advocate the theoretical and applied value of this theory and the usefulness of the ADQ in research and practice.

An appropriate point of departure might be the question of why we need another theory and questionnaire to describe, explain, and differentiate between anxiety and depression.

First, the presented theory allows for examining anxiety and depression in a general, not only clinical population. It seems to be very important in light of the latest meta-analysis (e.g., Ayuso-Mateos et al., 2010). Among others points, it demonstrated that the consequences of anxiety/depression for the general well-being in non-clinical populations when the main/full range of clinical criteria of anxiety/depression are not identified (e.g., low intensity of symptoms, low number of symptoms) are comparable with clinical populations. This implies the significance of analyzing the mechanisms of non-clinical forms of anxiety/depression and assessing them in the self-report instruments. As a review of the appropriate literature suggests, there are not many approaches and questionnaires that fulfill this need (see Fajkowska, 2013).

Second, the proposed theory represents a belief that non-clinical forms of anxiety/depression can be seen as relatively stable personality characteristics and reflects the newest results of the studies on cognitive and affective mechanisms in anxiety/depression (e.g., Eysenck and Fajkowska, 2017 for a review). Therefore, the questionnaire developed within it permits more precise hypotheses related to the origin of anxiety/depression to be formulated, supports the understanding of different consequences of functioning in these phenomena, and allows them to be evaluated on the basis of their maladaptive mechanisms (e.g., attentional, cf. Arditte and Joormann, 2014).

Third, the central finding in previous studies of anxiety and depression is the high degree of comorbidity that occurs between them (e.g., Gorman, 1996). Possible explanations of this co-occurrence relate to the poor discriminant validity of measures (e.g., Fox, 2008) and the fact that both phenomena are associated with negative affect (e.g., Watson, 2000), stressful life events (Naragon-Gainey and Watson, 2011), and impaired cognitive processes or a common biological/genetic diathesis (Watson and Kendall, 1989; Fox, 2008).

However, despite a set of nonspecific features, anxiety and depression are clearly not identical phenomena. The theory demonstrated here advocates that the differences between them might be best viewed through their heterogeneous and multilayered nature, adaptive functions, and relations with regulatory processes, positive affect, and motivation or complex cognitive processes (cf. Fajkowska, 2013). More precisely, differentiation should be improved by reducing the importance of overlapping features and by giving greater weight to distinctive aspects of these affective phenomena.

**Figure 1:**
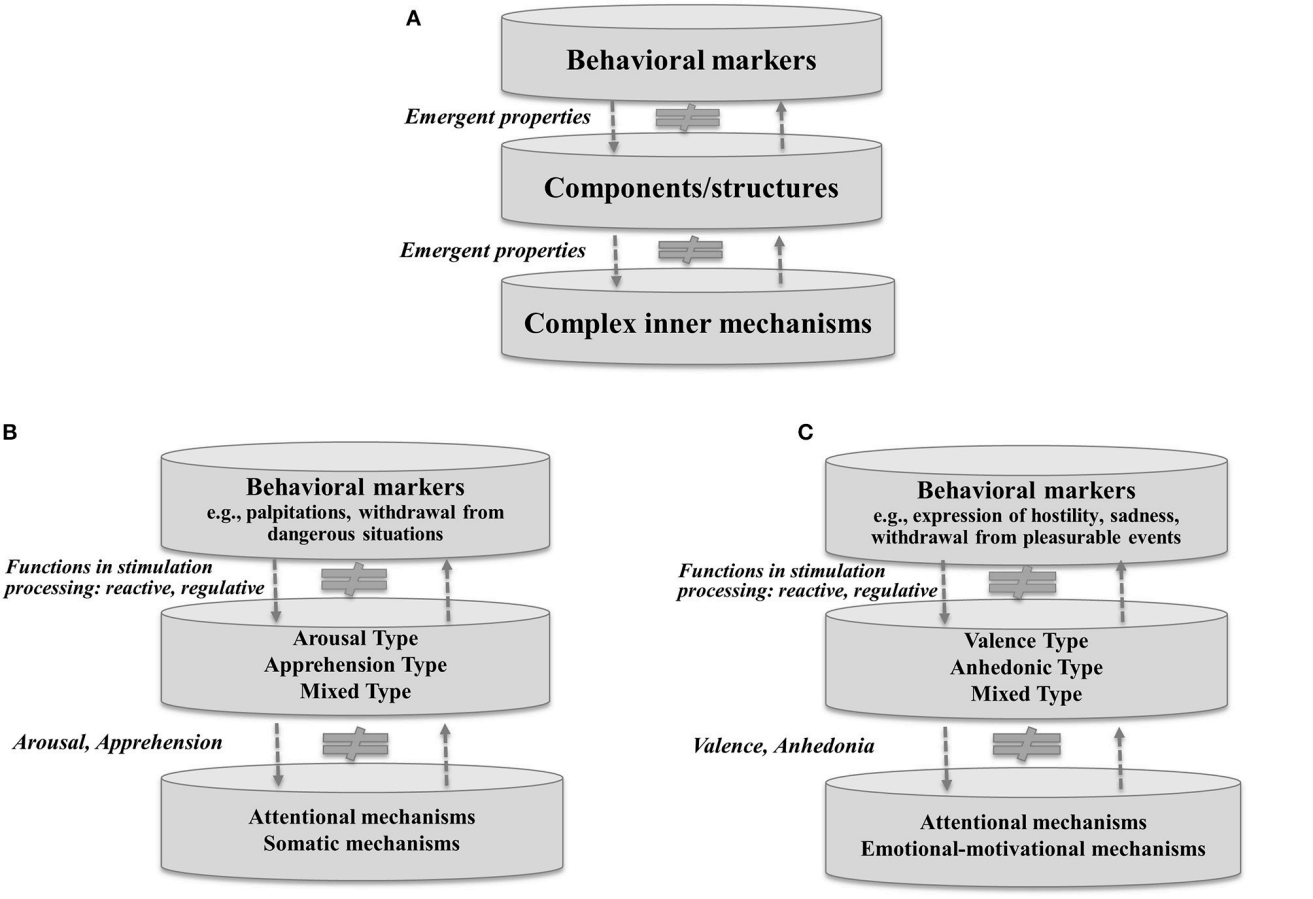
The organization of **(A)** personality trait/type, **(B)** anxiety types, **(C)** depression types according to the three-level hierarchy (cf. Fajkowska, 2013, 2015).

To meet all these points, Fajkowska (2013, 2015) suggests grouping anxiety and depression based on two criteria:

- The specificity of their structural composition; anxiety and depression are proposed to be seen as personality types embodying groups of traits (cf. Eysenck, 1998). Generally, both personality trait and type are defined as a hierarchical system, organized into three levels: complex inner mechanisms, components/structures, and behavioral markers (see Figure 1A). In this sense trait and type are equivalent, where types are structurally higher-order systems than traits, embracing a larger grouping of internal mechanisms and components than traits. Thus, the understanding of anxiety and depression as structurally complex personality types distinguishes this approach from the theory of Spielberger (1983), where anxiety is a homogeneous personality state or trait, and from the cognitive theories of depression (e.g., Beck, 1976; Bower, 1981; Teasdale, 1983) postulating its processual nature. In this approach a matched set of specific structural components and underlying processes are involved in building a particular type of anxiety or depression.
- The dominant functions (reactive or regulative) they play in stimulation processing (a transformation of arousal and activation, which arises as an effect of flowing stimulation, e.g., sensory, emotional, cognitive, leading to changes within different systems of the organism, e.g., motor, cognitive, or motivational). The dominant functions of a trait or type in stimulation processing might be considered as the emergent properties located between the level of structures and behavioral markers (see Figure 1A). In other words, these functions are rooted in structures and can be identified through overt reactions and behaviors (cf. Fajkowska, 2013). Traits/types with a reactive dominant (e.g., anxiety, Spielberger, 1983) inform about individual differences in the reception of flowing stimulation; they denote a high sensitivity or vigilance (e.g., sensory) to stimuli and rather automatic and immediate readiness to activity (reaction, behavior), and relate to energy expenditure (in a particular time range). For instance, the reactive function in anxiety can be identified through its associations with hypervigilance to threatening material or social evaluation (e.g., Eysenck, 2006). Traits/types with a regulative dominant indicate individual differences in energy expenditure (in a particular range of time) and more strategic than automatic/immediate directing and monitoring of the flowing stimulation, adequately to the organism's capacities for stimulation processing. For example, the regulative function in openness (Costa and McCrae, 1992) can be identified through its associations with creative and innovative strategies used to pursue one's goals (DeYoung, 2010). Additionally, the structural complexity of traits/types influences their controlling functions, which implies that different controlling functions might coexist in one trait (e.g., reactive-regulative in neuroticism, Eysenck, 1998). Thus, here anxiety and depression contribute to stimulation processing in that they relate to arousal, activation, and activity in different neurobiological and physiological systems (cf. Robinson and Compton, 2006). Therefore, it is further suggested that anxiety and depression can be differentiated according to the different functions they reveal in stimulation processing.

Although Fajkowska (2013) acknowledges that to some extent her categorization capitalizes on prior (neuro-)psychological models of emotion (cf. Heller, 1993a,b; Watson, 2000), these approaches seem to be rather categorical, while she suggests a dimensional typology (e.g., Eysenck, 1970; Strelau, 2014 p. 44–45). It enables the shared or separate structural components to be captured and to explain overlapping or distinctive functions in stimulation processing among types of anxiety and depression. Moreover, the current classification of anxiety and depression offered by the DSM-5 (American Psychiatric Association, 2013), although more dimensional than the previous DSM, seems not to be very supportive in solving some of the cardinal theoretical concerns in this area, e.g., specificity of the structure of affect or specificity of attentional biases in both anxiety and depression. Thus, a promising avenue to provide possible solutions to these concerns might be, as suggested here, an alternative grouping.

### Types of anxiety

#### Structural composition

The starting point for the identification of anxiety types is to point to relevant processes and mechanisms (the lowest level) that contribute to structures of anxiety types (the middle level), and to associate them with the relevant behavioral markers (the highest level; see Figure 1B).

- *Complex inner mechanisms*—With respect to the appropriate literature, Fajkowska (2013, 2015) assumes that somatic and cognitive processes are key for anxiety structuralization. The repetitive interactions among cognitive mechanisms (e.g., connected with attentional and working memory systems) and among somatic mechanisms (related to affective and motivational systems) lead to more integrated cognitive and somatic entities, from which emerge two essential elements that compose anxiety types: somatic-related arousal and cognitive-related apprehension (see Figure 1B).
- *Components/structures*—Thus, by interacting with each other, different levels of arousal and apprehension produce different types of anxiety at the level of structures (see Figure 1B). When the proportion between the degree of apprehension and degree of arousal is in favor of arousal, it suggests the *Arousal Type of anxiety*. When it is in favor of apprehension it produces the *Apprehension Type of anxiety*, and relatively equal (but high) levels of apprehension and arousal build the *Mixed Type of anxiety* [in previous publications (Fajkowska, 2013, 2015) used a misleading term, Balanced Type of anxiety, which suggested a positive type, but in fact it is composed of two disruptive elements].

Anxious arousal is described (cf. Watson et al., 1995; Watson, 2000) as being distinguished by symptoms of physiological hyperarousal and somatic tension, while anxious apprehension is primarily characterized by worry and verbal rumination, typically about future events (Barlow, 1991; Heller, 1993a,b; Heller et al., 1997; Heller and Nitschke, 1998). However, the relation between autonomic reactivity and anxious apprehension is not clear. Some studies report the connection of worrisome thoughts with elevated autonomic responsiveness (e.g., Nitschke et al., 1999), while others with autonomic rigidity (e.g., Thayer et al., 1996). The latter one seems to be more convincing, as worrisome thoughts are seen as a strategy for avoiding emotional arousal.

Panic attacks, phobias, high-stress states, and state anxiety as defined by self-report, behavioral, or physiological response systems would be covered by the Arousal Type (cf. Heller and Nitschke, 1998; Watson, 2000; American Psychiatric Association, 2013). It seems probable that the Apprehension Type would be characteristic of generalized anxiety states (GAD) and trait anxiety as identified by self-reports of anxious apprehension and worry on various questionnaires (cf. Heller and Nitschke, 1998; American Psychiatric Association, 2013). Theoretically, the Mixed Type might be identified among all the categories of anxiety mentioned above.

- *Behavioral markers*—The dominance of a particular component (arousal or apprehension) in a particular type of anxiety specifically determines the manner of stimulation processing, as well as patterns of response to stimulation across different response systems. More precisely, with reference to a review of the literature, it may be concluded that the typical patterns of attentional stimulation processing (that is reactions, behavioral acts) in the Arousal Type of anxiety are associated with (a) increased “early” attentional vigilance to threat (usually in clinical anxiety) and “later,” but unconscious, attentional avoidance of threat (usually in the non-patient group) (e.g., Calvo and Eysenck, 2000; Fox et al., 2002; Mathews and MacLeod, 2002; Wilson and MacLeod, 2003; Hock and Krohne, 2004; Heim-Dreger et al., 2006; Fisher et al., 2010); (b) elevated autonomic reactivity in the presence of threat (e.g., Sapolsky, 1992; Nitschke et al., 1999; Lovallo and Gerin, 2003; Hock and Krohne, 2004; Applehans and Luecken, 2006; Heim-Dreger et al., 2006; Fisher et al., 2010); and (c) right-hemisphere involvement in threatening stimuli processing (e.g., Heller et al., 1997; Compton et al., 2003; Engels et al., 2007; Mathersul et al., 2008). Accordingly, the typical patterns of stimulation processing in the Apprehension Type of anxiety are associated with (a) reduced attentional control and related impairment to the effectiveness of stimulation processing and avoidance of threatening stimuli (in clinical and nonclinical groups and trait anxiety; e.g., Laguna et al., 2004) (b) reduction in autonomic reactivity (e.g., Hoehn Saric et al., 1989; Borkovec and Ray, 1998) (c) impairment/inhibition of emotional processing, both on an attentional and physiological level (e.g., Stöber, 1998) and (d) left-hemisphere involvement in stimulation processing (cf. Tucker et al., 1978; Baxter et al., 1987; Swedo et al., 1989; Wu et al., 1991; Heller and Nitschke, 1997, 1998; Wagner, 1999; Fletcher and Henson, 2001; Nitschke and Heller, 2002; Hofmann et al., 2005).

#### Dominant functions

Thus, recognizing the presented above behavioral markers allows us to establish the dominant controlling function of each type: reactive rather than regulative in arousal anxiety (identified through more automatic stimulation processing related to attentional vigilance-avoidance, and also through elevated autonomic reactivity) and regulative rather than reactive in apprehension anxiety (identified through more strategic but ineffective stimulation processing related to reduced attentional control). It is assumed that the Mixed Type of anxiety is a functionally balanced type that represents a reactive-regulative function in stimulation processing.

### Types of depression

#### Structural composition

With reference to the identification of depression types (the middle level), the crucial mechanisms contributing to the formation of their structure are proposed (the lowest level) along with their related behavioral markers (the highest level; see Figure 1C).

- *Complex inner mechanisms*—In congruence with the relevant literature, Fajkowska (2013) proposed that cognitive and emotional-motivational processes are crucial in the formation of the structure of depression subtypes. The recurring interactions among cognitive mechanisms (connected with valence undersensitivity in attentional systems, e.g., Davidson et al., 1995), emotional mechanisms (linked with negative emotional experience, e.g., Beck et al., 1979) and the repetitive interactions among motivational mechanisms (associated with impaired control, anhedonia, reduction in response to reward-related stimuli, and a lack of positive reinforcement, e.g., Sloan et al., 2001), coupled with a deficit in approach behavior (e.g., Henriques and Davidson, 2000) lead to more integrated entities, from which in turn emerges more cognitive-related valence insensitivity and more emotion- and motivation-related anhedonia (see Figure 1C).
- *Components/structures*—Thus, dynamic interactions between the higher-ordered components—anhedonia and valence insensitivity—produce three types of depression: the *Valence Type of depression*, where the degree of valence insensitivity dominates the degree of anhedonia; the *Anhedonic Type of depression*, where the degree of valence insensitivity is dominated by the degree of anhedonia; and the *Mixed Type of depression* (previously named Balanced Type of depression, cf. Fajkowska, 2013), with a structure resting on a relative balance between (high levels of) the two components.

Thus, the valence insensitivity to stimulation is typical for non-melancholic forms of depression, while anhedonia is the key feature of melancholic depression (Heller and Nitschke, 1998; Watson, 2000), i.e., the inability to experience pleasure in all activities and a lack of responsiveness to pleasurable stimulation. However, melancholic and non-melancholic depression share many symptoms related to anhedonia, such as sadness, indecisiveness, feelings of guilt, and valence-related insensitivity such as inaccuracy in emotion recognition or inability to differentiate emotional states (Fajkowska, 2013).

All these types might be present in both nonclinical (depressed mood) and clinical forms of depression. The Valence Type embraces non-melancholic subtypes of depression, while the Anhedonic Type covers the melancholic subtypes (e.g., MDD) suggested by the DSM-5 (American Psychiatric Association, 2013). The Valence Type is treated here as an exogenous and state-like type, primarily connected with a biased cognitive system on account of the content or valence of stimulation. It is also connected with very high negative affectivity (see Fajkowska, 2013 for a review). The Anhedonic Type is relevant to an endogenous and trait-like type (cf. Rubino et al., 2009) and is primarily connected with impaired control in stimulation processing, motivational deficits, very high negative affect and very low positive affect (Watson, 2000). The Mixed Type of depression is a matter for future research.

- *Behavioral markers*—A review of the literature allows for the conclusion that specific patterns of attentional stimulation processing in the Valence Type depression are related to (a) attentional avoidance reflected in valence insensitivity to emotional and social material (e.g., Gotlib et al., 2000; Watson, 2000; Fox, 2008), and (b) increased right-hemisphere activity in stimulation processing (e.g., Heller and Nitschke, 1997; Parker et al., 1999; Nitschke et al., 2001; Sato et al., 2001; Tembler and Schüßler, 2009; Hecht, 2010). On the basis of both theoretical and empirical evidence, it turns out that specific patterns of stimulation processing in the Anhedonic Type of depression are related to (a) impaired attentional control, or sustained attention over positive as well as negative material (e.g., Bargh et al., 1988; Gotlib and MacLeod, 1997; Westra and Kuiper, 1997; Egeland et al., 2003; Marszał-Wiśniewska and Fajkowska-Stanik, 2005; Withall et al., 2009; Bourke et al., 2010), and (b) decreased left-hemisphere activity in stimulation processing (e.g., Bench et al., 1992; Heller, 1993a; Bruder, 1995; Hecht, 2010; Schock et al., 2011).

#### Dominant functions

Again, the identification of the above presented behavioral markers allowed the dominant controlling functions of each subtype to be established: reactive rather than regulative in valence depression (identified through more automatic stimulation processing related to attentional avoidance of stimuli), and regulative rather than reactive in anhedonic depression (identified through more strategic but ineffective stimulation processing related to reduced attentional control and inability to sustain attention over stimulation). In the Mixed Type of depression the mixed, reactive-regulative function over stimulation processing is postulated.

### Operationalization of the anxiety and depression types

**Figure 2:**
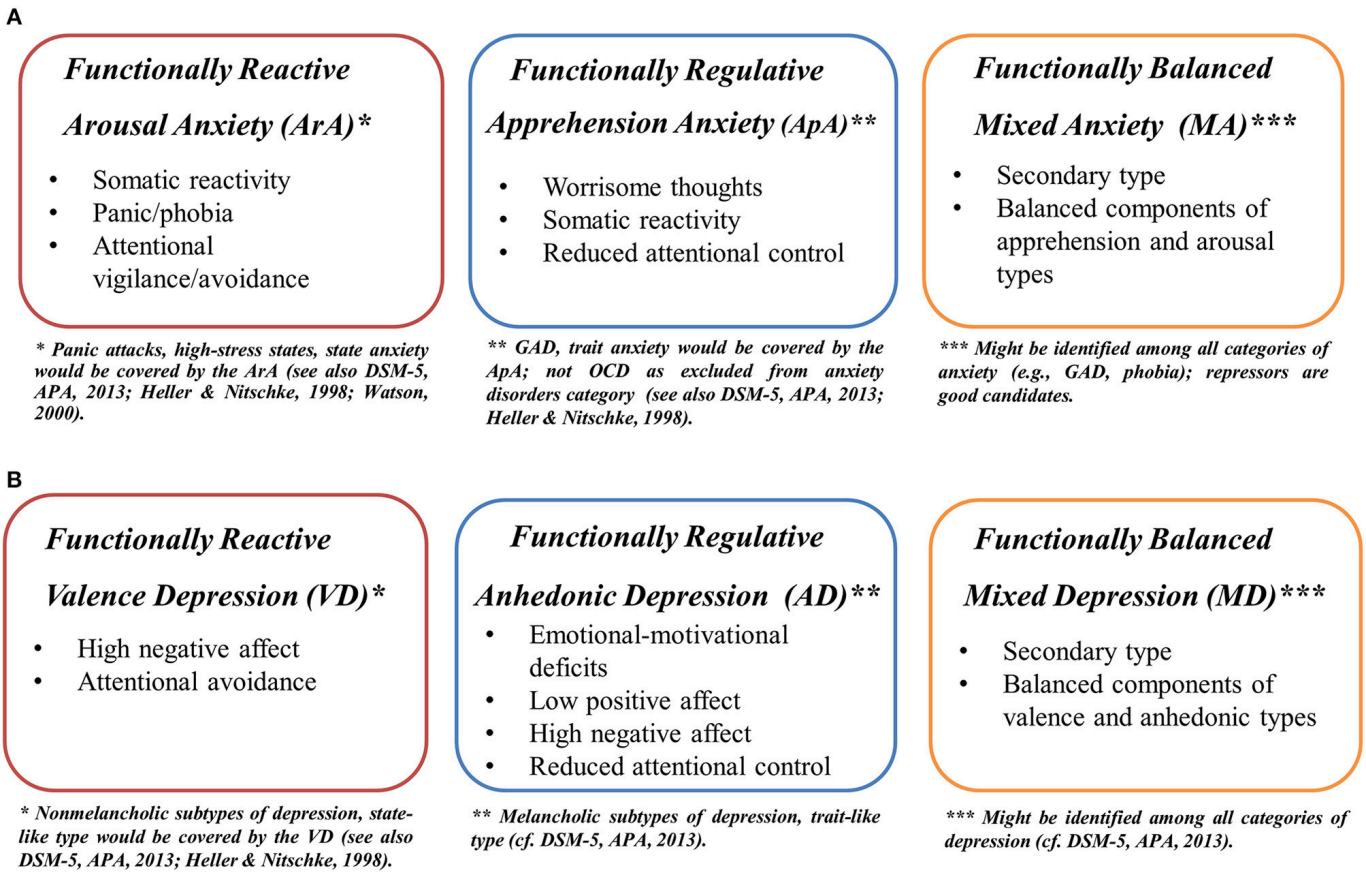
Operationalization of **(A)** anxiety types, **(B)** depression types (cf. Fajkowska, 2013, 2015).

The evidence discussed here provides important information that has contributed to the development of precise definitions of anxiety types according to their structural components and functions in controlling stimulation (see Figure 2A):

Reactive *Arousal Type of anxiety* is composed of:

- *Somatic Reactivity*—Elevated autonomic reactivity, psychophysiological hyperarousal and somatic tension (e.g., trembling hands, palpitations, sweating, gastric problems, shortening breath) in the presence of actual or anticipated threat or negative stimulation.
- *Panic/Phobia*—Presence of panic symptoms, distress (e.g., related to fear of heights or new situations or objects) and phobias (e.g., social).
- *Attentional Vigilance/Avoidance*—“Early” attentional vigilance toward threat (very fast identification of threat or negative social signals appearing in the attentional field), usually in clinical forms of anxiety, and “later” attentional avoidance of this threat (rather instinctive than intentional withdrawal from dangerous and threatening situations present in the attentional field for some time), usually in the non-patient groups.

Regulative *Apprehension Type of anxiety* is composed of:

- *Worrisome thoughts*—Referring to physical, emotional, or symbolic threats to the self; they relate to social evaluation of one's behavior or competences; sometimes their content may include general world problems;
- *Somatic Reactivity*—Elevated autonomic reactivity in the presence of threat, or because of worrisome thoughts; it goes with reduced capacities of emotional processing on autonomic and somatic levels.
- *Attentional control*—Reduced attentional control, that relates to difficulties in attentional (a) shifting, (b) focusing, and (c) disengagement from negative experiences; (d) it undergoes distracting thoughts, and (e) reveals itself in impaired inhibition in processing of negative emotional material, connected with failure or negative experiences or events;

*Mixed Type of anxiety* represents balanced apprehension and arousal elements and balanced reactive and regulative functions of stimulation processing. Speculatively, it covers specific patterns of stimulation processing of both primary types (arousal and apprehension) and their activation might be situation-dependent.

Figure 2B summarizes definitions of the three types of depression according to their structural components and the functions they play in stimulation processing.

Reactive *Valence Type of depression* goes with:

- *Negative Affect*—Manifested in increased level of anxiety, tension, hostility, anger, sadness, self-sensitivity, and social avoidance.
- *Attentional Avoidance*—Identified through (a) valence insensitivity to emotional and social material, i.e., delayed or constricted attention allocation toward emotional material, inaccurate recognition of emotional material regardless of its (positive or negative) content and (b) insensitivity to social material, including emotions appearing in the social context.

Regulative *Anhedonic Type of depression* includes:

- *Emotional-Motivational Deficits*—Revealed in (a) the inability to experience pleasure and decreased reactivity to pleasurable things and events, (b) difficulties in goal achievement and loss of interest in pursuing goal-directed activities, and (c) failure in delivering sufficient pleasure or reward following approach behaviors.
- *Positive Affect*—Extremely low positive affect; very low level of positive emotions, e.g., self-confidence, happiness, hope, or satisfaction.
- *Negative Affect*—Extremely high negative affect; very high level of negative emotions, e.g., sadness, guilt, shame, sense of loss, disappointment, anxiety, and loneliness.
- *Attentional Control*—Impaired attentional control, indicating (a) decrement in sustained vigilance to emotional material, (b) slower and inaccurate response to emotional material (e.g., slower reactions to positive material and inaccurate recognition of negative material), (c) inability to sustain effort in processing emotional material (regardless of its valence), and (d) difficulties in attentional focusing.

*Mixed Type of depression* is defined through the relative balance of the valence and anhedonia elements, and balanced reactive and regulative functions of stimulation processing. It most probably comprises specific patterns of stimulation processing of both primary (valence or anhedonic) types, and their activation might depend on the specific situation.

Even though some of the structural components overlap across various affective types, they do not always mean the same. Somatic reactivity is a component very specific for anxiety (cf. Watson, 2000), thus it appears in both types of anxiety. However, it has different causes and expressions. In arousal anxiety it is a primary element, while in the apprehension type it is not a crucial one, it is caused by worrisome thoughts and is rather expressed as reduced somatic reactivity. Next, attentional control is present in both regulative types, i.e., apprehension anxiety and anhedonic depression. In apprehension anxiety it appears as an effect of worrisome thoughts and primarily indicates impaired inhibition functioning, while in anhedonic depression it appears as an effect of emotional-motivational deficits and negatively influences prolonged and sustained attention. Finally, negative affect is a part of the structure in both depression types. Nonetheless, hostility and anger are typical for valence depression, while for anhedonic depression it is guilt and shame.

With reference to dominant functions in controlling stimulation processing, one should expect similarities in patterns of stimulation processing (e.g., in attentional processing) across types, i.e., reactive types, regulative types and functionally balanced types, and differences within types, i.e., between reactive and regulative types (see Figure 2).

### Anxiety and depression questionnaire (ADQ)

A psychometric study was conducted with the aims of revising the postulated anxiety and depression types (Fajkowska, 2013, 2015) and of constructing an instrument, the Anxiety and Depression Questionnaire (ADQ), which corresponds to the six affective types. Consequently, we proposed four scales of the ADQ to directly measure arousal anxiety (ADQ-ArA), apprehension anxiety (ADQ-ApA), valence depression (ADQ-VD), and anhedonic depression (ADQ-AD). However, in line with the theory (Fajkowska, 2013, 2015), the mixed types of anxiety and depression should be regarded, respectively, as the ratio of arousal anxiety to apprehension anxiety and valence depression to anhedonic depression, and they have the status of secondary types (cf. balance of the nervous processes as a secondary trait/scale, Strelau et al., 1999).

## General plan of research and analysis

The elaboration and development of the ADQ has been divided into construction (Study 1) and validation (Study 2) stages.

The aim of Study 1 was the generation of items and delivering psychometric characteristics of the preliminary version of the ADQ. Thus, for four scales of the ADQ we evaluated (a) the discriminatory power of items to answer the question to what degree the particular test positions differentiate among individuals; (b) confirmatory factor analysis to test the theoretically suggested structure of the particular ADQ scales; (c) intercorrelations of subscales within the scales of the ADQ to check if theoretically predicted relations among them are supported by empirical data; and (d) internal consistency for all scales of the ADQ to measure the extent to which all of the items of a certain scale measure the same latent variable. With these data we made appropriate corrections to propose the final form of the ADQ.

The Study 2 was organized around demonstration of the quantitative description of items and scales of the ADQ, reliability and validity of the ADQ, and verification of the assumed position of the affective types among other personality characteristics.

## Construction stage—study 1

Extensive research aimed at constructing the ADQ consisted of the generation of items, linguistic evaluation of items, evaluation of content validity, on-line administration of the questionnaire to respondents, and elaboration of the experimental version of the questionnaire based on the results from testing of discriminatory power of items, confirmatory factor analysis, intercorrelational analysis of subscales, and internal consistency.

## Materials and methods

### Generation of the ADQ items

The first development stage involved the generation of items (experts, *n* = 4; Ph.D. students of psychology, *n* = 4) that will form the four scales of the ADQ. This generation was guided by methodological requirements underlying the construction of personality inventories (cf. Zawadzki, 2006) and operational definitions of each affective type. Additionally, about five percent of the total number of items was taken, mostly in slightly modified versions, from other inventories [e.g., Attentional Control Scale (ACS), (Fajkowska and Derryberry, 2010), Mood and Anxiety Symptom Questionnaire (MASQ), (Watson, 2000), The Penn State Worry Questionnaire, (Meyer et al., 1990)].

The linguistic analysis of items, as well as the assessment of the content validity (sorting items into types and subscales, experts, *n* = 4), led to the development of an item pool for each scale. Arousal anxiety consists of 64 items grouped into subscales of Somatic Reactivity (SR, 35 items), Panic/Phobia (PP, 18), and Attentional Vigilance/Avoidance (AVA, 11), while apprehension anxiety has 89 items in subscales of Worrisome Thoughts (WT, 22), Attentional Control (AC, 44), Attentional Avoidance (AA, 13), and Somatic Reactivity (rSR, 10; “r” means that items indicate reduced somatic activity). It should be noted that although Attentional Avoidance is not mentioned in the definition of apprehension anxiety, we introduced this scale experimentally as some studies report significant connections between attentional avoidance and apprehension (Laguna et al., 2004).

Valence depression consists of 71 items clustered around Negative Affect (NA, 50) and Attentional Avoidance (AA, 21), and for anhedonic depression there are 139 statements in four subscales of Emotional-Motivational Deficits (EMD, 87), Positive Affect (PA, 12), Negative Affect (NA, 26), and Attentional Control (AC, 14). Attention was paid during all stages of item generation to keep a balanced keying within each of the scales.

### Participants

Since the theory assumes that affective types are personality traits rather than purely clinical disorders, the study was conducted on two general, non-clinical samples. Both samples matched the demographic structure of the Polish population.

The first sample (*N* = 1,109) consisted of 546 males (49.2%) and 563 females (50.8%) with a mean age of 39.19 (*SD* = 13.59; range 18–65 years). Participants filled out the two scales of the ADQ measuring anxiety types: ADQ-ArA and ADQ-ApA.

39.7% held university degrees, 36.6% finished high school, 15.9% vocational school, and 7.8% elementary school. The participants were of various professional and educational backgrounds, including university students, high-school students, working people, white-collar workers, part-time workers, unemployed, and pensioners. 49.3% of the participants came from villages and small towns, 20% from cities, and 30.7% from big cities and metropolises. 26.1% reported that they had experienced anxiety disorders in the past, and 15.3% suffered from anxiety at the moment of the study. Moreover, 10.1% of respondents benefited from psychological and psychiatric help, and 3.4% were hospitalized because of severe anxiety. The respondents specified different phobias, panic attacks, separation anxiety, and anxiety coexisting with other disorders and states (e.g., depression, traumatic experiences and addictions, reaction to rape, violence, and unsuccessful social and family relations).

The second sample contained 1,086 participants (549 [50.6%] females and 537 [49.4%] males) who were on average 39.01 years old (*SD* = 13.33; range 18–65 years). They completed the ADQ-VD and the ADQ-AD scales, assessing depression types.

36.6% of the participants completed university education, 36.1% finished high school, 20.3% vocational school, and the remaining 7% elementary school. They represented different professions and schools, including university students, high-school students, working people, white-collar workers, part-time workers, and pensioners. 48.8% of the sample represented inhabitants of villages and small towns, 20.4% of cities, and 30.8% of big cities and metropolises. 22.9% of individuals acknowledged suffering from depression in the past and 12% in the present. In addition, 12.3% received help from psychological and psychiatric services, and 4.5% underwent hospitalization because of depression. They reported reactive depression (e.g., because of loss of a close person or job, difficult family relationships), bipolar depression, major depression (with dominating sadness, lack of values in life, low self-esteem, suicidal thoughts) and depression concomitant with other disorders (e.g., alcoholism, psychosis, borderline personality).

### Procedure

This study was approved by the ethics committee of the Institute of Psychology, Polish Academy of Sciences. Participants provided their written informed consent before the on-line procedure was activated. Participants were recruited from an on-line research panel and every person who completed the full procedure on-line received points that were exchangeable for rewards. The order of the questionnaires was randomized across subjects. Altogether they contained 153 (anxiety questionnaires) and 210 (depression questionnaires) agree-disagree items that allow the assessment of arousal anxiety and apprehension anxiety in the first sample, and valence and anhedonic depression in the second sample.

### Scoring

All of the items included in the arousal anxiety (ADQ-ArA) and valence depression (ADQ-VD) scales are summed to score (1 point per diagnostic item Agree/Disagree), though extra calculations are required for the AC subscales of apprehension anxiety (ADQ-ApA) and for the anhedonic depression (ADQ-AD) scales. To calculate the scores on these scales, the obtained score should be subtracted from the maximum possible score in the given scale, because we are interested in evaluating decreased attentional control (while the items measure the strength of attentional control).

### Statistical analysis

We performed analyses on the discriminatory power of items (Youle's Phi coefficients; ϕ, Phi), confirmatory factor analysis (CFA), intercorrelations of subscales (Pearson's *r*) and internal consistency (Cronbach's α coefficients) in the first and second sample separately.

## Results

### Discriminatory power of items

Discriminatory power of items was used as the criterion for excluding the preliminarily selected items from the ADQ. For all scales of the ADQ, the number of items that was kept depended on their Youle's Phi coefficients (ϕ,). We decided to apply the value ϕ ≥ 0.30 as it is a correlation between the item score and the overall scale score reduced by this item, which is usually lower than the correlation between item and the total scale score, and values 0.30 and above indicate good and very good discrimination (see Drwal and Brzozowski, 1995). The number of remaining items in each subscale of each ADQ scale was sufficient for further statistical analysis (ArA = 42, ApA = 62, VD = 36, AD = 69).

### Confirmatory factor analysis

Confirmatory factor analysis (CFA) was used to verify the factor structure of the set of introduced variables (Kline, 2005). More precisely, we tested the theoretically suggested structures of the affective types. We expected that the models including the number of factors derived from the theory will show a better fit than other (e.g., one-factor) models. The analyses were performed in Mplus (Muthén and Muthén, 1998), which enables models of binary data to be built (see Górniak, 2000; Hox, 2002). Because χ^2^ values for the models fit across all scales of the ADQ were significant (suggesting a lack of fit between the hypothesized models and the data) and due to the sensitivity of χ^2^ in large samples, other fit indices were assessed and reported, namely: Comparative Fit Index (CFI); Root Mean Square Error of Approximation (RMSEA); Tucker Lewis Index (TLI) (Kline, 2005).

In arousal anxiety (ADQ-ArA) we compared a one-factor solution with a three-factor solution. The fit indexes reflected the improvement in fit of the three-factor model [RMSEA = 0.051; CFI = 0.92; TLI = 0.92; χ^2^/df = 3.93; $\chi_{\left(816,\;\text{N}\;=\;1,109\right)}^{2}$ = 3211.13, *p* < 0.001] over the alternative one [RMSEA = 0.054; CFI = 0.91; TLI = 0.91; χ^2^/df = 4.24, $\chi_{\left(819,\;\text{N}\;=\;1,109\right)}^{2}$ = 3474.33, *p* < 0.001]. All items loaded significantly onto their respective factors (loadings ranging from 0.66 to 0.84 on the SR subscale, from 0.43 to 0.87 on the PP subscale, and between 0.29 and 0.86 on the AVA subscale). None of the test positions were eliminated from further analysis.

To assess the factor structure of the ADQ-ApA, the fit of one-factor and four-factor models was examined. As predicted, the model fit for the four factors [RMSEA = 0.055; CFI = 0.87; TLI = 0.87; χ^2^/df = 4.33; $\chi_{\left(1,823,\;\text{N}\;=\;1,109\right)}^{2}$ = 7908.17, *p* < 0.001] was better than for one factor [RMSEA = 0.057; CFI = 0.86; TLI = 0.85; χ^2^/df = 4.56; $\chi_{\left(1,829,\;\text{N}\;=\;1,109\right)}^{2}$ = 8354.69, *p* < 0.001]. Given that the four-factor model did not reach the fit parameters (CFI, TLI) over 0.90, items with the lowest factor loadings were removed. Indeed, the fit indexes improved [RMSEA = 0.053; CFI = 0.92; TLI = 0.92; χ^2^/df = 4.16; $\chi_{\left(1,420,\;\text{N}\;=\;1,109\right)}^{2}$ = 5918.256, *p* < 0.001]; however, the left items were not differentiated in their meaning as the factor analysis favors items similar in their content. Thus, for the sake of better psychological and theoretical rationality of items, we decided to keep all of them for subsequent elaboration of the ADQ-ApA. As a result, factor loadings for the WT items ranged from 0.49 to 0.89, for the AC varied from 0.36 to 0.85, for the AA extended from 0.43 to 0.86, and for the rSR oscillated from 0.47 to 0.87.

In case of the ADQ-VD we tested one-factor and two-factor models. Contrasting with the first model, the latter one had a good fit [respectively, RMSEA = 0.071; CFI = 0.88; TLI = 0.87; χ^2^/df = 6.46; $\chi_{\left(816,\;\text{N}\;=\;1,109\right)}^{2}$ = 3211.133, *p* < 0.001; and RMSEA = 0.057; CFI = 0.92; TLI = 0.92; χ^2^/df = 4.56; $\chi_{\left(594,\;\text{N}\;=\;1,086\right)}^{2}$ = 3838.70, *p* < 0.001]. The lowest factor loading was 0.58 and the highest 0.86 on the NA factor, while on the AA factor we had a range of factor loadings from 0.59 to 0.84.

The hypothetical model of the structure of the ADQ-AD was grounded on four factors, but we examined three competing models of one-factor, three-factors (EMD, NA—boosted by the items of PA, treated as reversed, and of AC), and four-factors (EMD, NA, PA, and AC). The findings suggested that the four-factor solution was the best one [RMSEA = 0.049; CFI = 0.93; TLI = 0.93; χ^2^/df = 3.63; $\chi_{\left(2,271,\;\text{N}\;=\;1,086\right)}^{2}$ = 8254.56, *p* < 0.001] compared to the one- and three-factor models [respectively, RMSEA = 0.051; CFI = 0.92; TLI = 0.92; χ^2^/df = 3.82; $\chi_{\left(2,277,\;\text{N}\;=\;1,086\right)}^{2}$ = 8717.44, *p* < 0.001; and RMSEA = 0.050; CFI = 0.92; TLI = 0.92; χ^2^/df = 3.71; $\chi_{\left(2,274,\;\text{N}\;=\;1,086\right)}^{2}$ = 8441.85, *p* < 0.001]. The final assessment concerned the factor loadings. For the EMD they ranged from 0.64 to 0.89, for the NA from 0.74 to 0.93, for the PA from 0.63 to 0.93, and for the AC from 0.32 to 0.91. Two items of the AC subscale with the lowest factor loadings were kept after linguistic correction.

### Intercorrelations of subscales

With reference to the relevant data (Fajkowska, 2013, for a review see: Watson, 2000), we expected positive correlations (Pearson's *r*) among the ADQ-ArA subscales. Indeed, the analyses revealed a positive relationship between the SR subscale and the PP subscale (0.32, *p* < 0.01), the SR and the AVA subscales (0.28, *p* < 0.01), and the PP and the AVA subscales (0.18, *p* < 0.01).

Supported by the neuropsychological models of emotions (e.g., Heller, 1993a,b; Heller and Nitschke, 1998; for a review see: Fajkowska, 2013) in the case of the ADQ-ApA, we predicted (a) positive relations among subscales WT, AA, and rSR, and (b) negative relations between the AC subscale and WT, AA, and rSR subscales. The obtained results (Pearson's *r*) confirmed these speculations to some extent. As predicted, the WT subscale correlated positively with the AA subscale (0.23, *p* < 0.01) and negatively with the AC subscale (−0.44, *p* < 0.01). However, contrary to expectations rSR correlated negatively with the WT and AA subscales (−0.44, *p* < 0.01 and −0.23, *p* < 0.01, respectively). Many psychophysiological studies reveal that anxious apprehension, unlike other anxious states, are not associated with a greater response of the autonomic system but rather with autonomic rigidity (e.g., Hoehn Saric et al., 1989; Thayer et al., 1996). However, in the long-term perspective it was observed that in addition to worry, physical symptoms and elevated physiological arousal often accompany anxious apprehension (e.g., Nitschke et al., 1999; Laguna et al., 2004). These outcomes are in line with our findings. Thus, the elevated autonomic responsiveness relates to decreased attentional control (positive correlation of rSR with AC, 0.32, *p* < 0.01).

With regards to the appropriate data presented in the literature (see Fajkowska, 2013 for a review), we anticipated a positive but weak correlation between subscales of the ADQ-VD, i.e., between the NA and AA subscale. The findings (Pearson's *r*) are in accordance with predictions (0.35, *p* < 0.01). The attentional insensitivity (or avoidance) to valence of emotional and social material is connected with negative affectivity, which is typical for e.g., anxiety or non-melancholic types of depression (see Fajkowska, 2013).

Turning to the relations among subscales of the ADQ-AD, the results of other studies suggest that we should expect positive and moderate associations between EMD and NA, as well as AC and PA, and negative ones between EMD, AC, and PA (see Fajkowska, 2013), and non-significant relations between NA and PA, defined here as affective traits (see Watson, 2000). The collected data (Pearson's *r*) partially confirmed these expectations. It was found that negative affect relates negatively to positive affect (−0.59, *p* < 0.01). The bipolar relation between NA and PA reflected in this study might be explained by the fact that both affects were explored with items representing very intensive negative and positive states. In other words, a high or very high level of NA implies a low or very low level of PA, and vice versa (Watson and Tellegen, 1985; Watson, 2000).

The data revealed a positive relation between EMD and NA (0.71, *p* < 0.01) and a negative relation between EMD and PA (−0.65, *p* < 0.01), which Watson (2000) also documented in his research. Motivational deficits (among others understood as the loss of appetitive behaviors and interest in pursuing goal-directed activities) relate to the very specific for this type of depression (a) marked reduction in experiencing pleasure and extremely low PA, and (b) high NA, nonspecific for it (Watson, 2000).

In addition, several studies have shown that effective attentional control is subjected to positive mood, while negative affect has an adverse effect on it (see Fajkowska, 2013 for a review), which is congruent with the results of our study (0.65, *p* < 0.01 and −0.63, *p* < 0.01, correlations between AC and PA, AC and NA, respectively). The association between EMD and AC should be negative, and it was (−0.63, *p* < 0.01). There is a conflict between the intentional and effortful, effective attentional control (cf. Fajkowska and Derryberry, 2010) and emotional-motivational deficits—difficulties in pursuing goals and tasks, putting effort into realizing them, and troubles in undertaking and initiating activities.

### Internal consistency

The results showed high Cronbach's alphas for all scales of the ADQ (42-item ADQ-ArA = 0.93; 62-item ADQ-ApA = 0.91; 36-item ADQ-VD = 0.93; 69-item ADQ-AD = 0.82). High and moderate internal consistency was also observed across all subscales of each ADQ scale, namely: arousal anxiety (ADQ-ArA: 19-item SR = 0.91; 15-item PP = 0.86; 8-item AVA = 0.53), apprehension anxiety (ADQ-ApA: 16-item WT = 0.90; 29-item AC = 0.91; 8-item AA = 0.53; 9-item rSR = 0.79), valence depression (ADQ-VD: 21-item NA = 0.92; 15-item AA = 0.87), and anhedonic depression (ADQ-AD: 33-item EMD = 0.95; 12-item PA = 0.91; 12-item NA = 0.92; 12-item AC = 0.76). The two shortest subscales—AVA from the ADQ-ArA and AA from the ADQ-ApA—showed the lowest internal consistency.

### Final remarks

Based on the results from the analysis of discriminatory power of items and from the CFA, we proposed an experimental version of the ADQ. However, a few final corrections were made. Some items were excluded (e.g., with the lowest factor loadings), some linguistically corrected, and some moved to different subscales, and the subscale of AA was removed from the ADQ-ApA due to having very weak psychometric parameters (results cumulated from all analyses). The intercorrelational analysis among the subscales of the ADQ-ApA revealed that the rSR subscale is not valid. Thus, supported by these results and the appropriate results from the other studies (e.g., Laguna et al., 2004), we decided to transform this subscale into one assessing elevated somatic reactivity in apprehension anxiety.

Moreover, we added some filler items to the arousal anxiety (ADQ-ArA) and valence depression (ADQ-VD) scales to balance keying within each of the scales. Generally, it was not always possible to find a sufficient number of well-balanced items.

## Validation stage—study 2

The second stage was designed as a validation study in order to ensure that the developed questionnaires of affective types are valid and reliable; the second aim was to verify the theoretically postulated location of the affective types among other personality constructs. Consequently, we report item and scale statistics, present evidence on the intercorrelations between subscales of each scale of the ADQ, and also assess the content and construct validity (that is factor structure, convergent, and divergent validity with well-established measures of related personality constructs, and theory-consistent group differences) and the stability and reliability of the questionnaires.

## Materials and methods

### Four scales of the ADQ

The final form of the ADQ (please see the Supplementary Material) is composed of four scales, directly measuring arousal anxiety, apprehension anxiety, valence depression, and anhedonic depression, and indirectly measuring mixed types of anxiety and depression. There is a dichotomous response format for all items (Agree/Disagree). The scoring system is identical as previously described.

ADQ-Arousal Anxiety (ADQ-ArA): 45 items (including 4 fillers), 3 subscales

- Somatic Reactivity (SR, 22 items); e.g., When something scares me, I feel a sudden attack of heat or cold.
- Panic/Phobia (PP, 14 items); e.g., I do not panic, even in the face of threats and dangers (reversed).
- Attentional Vigilance/Avoidance (AVA, 5 items); e.g., When I notice a potential threat, I automatically withdraw from the given situation.
- Fillers (4 items), e.g., I enjoy reading books.

ADQ-Apprehension Anxiety (ADQ-ApA): 48 items, 3 subscales

- Worrisome Thoughts (WT, 14 items); e.g., I am not in the habit of worrying excessively (reversed).
- Attentional Control (AC, 23 items); e.g., I cannot concentrate on a difficult task if there are noises around.
- Somatic Reactivity (SR, 11 items); e.g., When facing danger, I often feel like my legs “turn to jelly.”

ADQ-Valence Depression (ADQ-VD): 40 items (including 4 fillers), 2 subscales

- Negative Affect (21 items); e.g., I often get angry.
- Attentional Avoidance (15 items); e.g., I find it difficult to notice that someone is sad.
- Fillers (4 items); e.g., I prefer to travel by car rather than by train.

ADQ-Anhedonic Depression (ADQ-AD): 64 items, 4 subscales

- Emotional-Motivational Deficits (EMD; 31 items); e.g., I can start new things without difficulty (reversed).
- Positive Affect (PA; 13 items); e.g., I often smile honestly and joke.
- Negative Affect (NA; 12 items); e.g., I often feel sad.
- Attentional Control (AC; 8 items); e.g., Emotional events distract me so much that I later have trouble concentrating.

### Participants

Table 1 demonstrates the socio-demographic characteristics of the validation, non-clinical sample (*N* = 1,632). The sample matched the demographic structure of the Polish population. Participants who provided an unusually high number of identical answers on any questionnaire were removed from analyses. This procedure was used for each questionnaire separately. For ADQ and EPQ-R we removed participants with *M*+2*SD* of identical answers, and for the remaining questionnaires we removed those who had zero variance in their answers, as the *M*+2*SD* procedure turned out to be ineffective. As a result, from 4 to 11.8% (*M* = 7.6%) of the participants were removed from the original sample (*N* = 1,632), hence the differences in *N*s across analyses.

**Table 1:** Socio-demographic characteristics of the sample.

**Age**		**Sex**		**Highest completed education**				**Residence**	
***M***	***SD***	**Male**	**Female**	**University Degree**	**High school**	**Vocational school**	**Elementary school**	**Rural**	**Urban**
39.00	13.10	783 (48%)	849 (52%)	38.8%	49.3%	9.1%	2.8%	48.6%	51.4%

*M, mean; SD, Standard deviation*.

Except demographic questions, participants were asked about whether they suffered (now or in the past) from anxiety or depression, and if “yes” they were questioned about psychotherapy, pharmacotherapy, hospitalization, professional diagnosis, and causes of these disorders. Respectively, 23.5 and 19.9% of individuals admitted that they experienced anxiety or depression disorders in the past. 13.3% of them reported that they had suffered from anxiety and 11.1% from depression in the moment of the study. Furthermore, owing to anxiety or depression, correspondingly 9.5 and 11.7% of respondents reported having used psychological and psychiatric help. 4.1 and 5% had been hospitalized because of severe anxiety or depression, respectively. The participants stated different phobias, panic attacks, separation anxiety, and anxiety coexisting with other disorders and states, reactive depression, bipolar depression, major depression, and depression associated with other disorders.

### Procedure

The ethics committee of the Institute of Psychology, Polish Academy of Sciences approved this on-line study and the consent procedure elaborated to it. The procedure of participants' recruitment and data collection were the same as in Study 1.

Respondents across two separate sessions completed a battery of on-line self-report techniques, randomized across subjects, and across sessions:

- Four scales of the ADQ.
- *State–Trait Anxiety Inventory* (STAI), which consists of two 20-item subscales: one measuring state anxiety and the other measuring trait anxiety (Spielberger, 1983; Wrześniewski and Sosnowski, 1996).
- *Beck Depression Inventory* (BDI-II), composed of 21 questions assessing intensity of depressive symptoms (Beck et al., 1996; Zawadzki et al., 2009).
- *Behavioral Inhibition System/Behavioral Approach System scales* (BIS/BAS scales)—the 24-item measure assessing dispositional BIS and BAS sensitivities. It includes three BAS-related scales: BAS Drive, BAS Fun Seeking, and BAS Reward Responsiveness (Carver and White, 1994; Müller and Wytykowska, 2005).
- *Positive and Negative Affect Schedule—Expanded Form* (PANAS-X) a 60-item questionnaire comprising two higher level scales reflecting the valence of affect, that is Positive Affect (PA) and Negative Affect (NA) scales, and 11 lower level scales reflecting their specific content: Fear, Sadness, Guilt, Hostility (Basic Negative Emotion Scales); Joviality, Self-Assurance, Attentiveness (Basic Positive Emotion Scales); Shyness, Fatigue, Surprise, and Serenity (Other Affective States) (Watson and Clark, 1994; Fajkowska and Marszał-Wiśniewska, 2009).
- *Cognitive Emotion Regulation Questionnaire* (CERQ), a multidimensional technique constructed in order to identify the cognitive emotion regulation strategies someone uses after having experienced negative or traumatic events. It contains 36 items measuring nine different cognitive coping strategies, including four non-adaptive: Self-blame, Rumination, Catastrophizing, and Other blame and five adaptive ones: Acceptance, Positive refocusing, Refocus on planning, Positive reappraisal, Putting into perspective (Garnefski et al., 2002; Marszał-Wiśniewska and Fajkowska, 2010).
- *Attentional Control Scale* (ACS)—The 20-item ACS measures the ability to focus perceptual attention, switch attention between tasks, and flexibly control thought (Derryberry and Reed, 2002; Fajkowska and Derryberry, 2010).
- *Eysenck Personality Questionnaire Revised—Short Version* (EPQ-R [S])—short version contains 48 items from the full EPQ-R. Includes scales: Psychoticism (P), Extraversion (E), Neuroticism (N), and Lie (L) (Eysenck et al., 1985; Jaworowska, 2011).

### Statistical analysis

To provide a general statistical description of items and scales of the ADQ we elaborated means, standard deviations, Cronbach's α coefficients, the discriminatory power of items (Youle's Phi coefficients; ϕ), intercorrelations of subscales (Pearson's *r*) on the total sample, the means, standard deviations, and *t*-tests showing sex differences, and the prevalence of affective types in women and men.

The content validity of the ADQ was assessed with inter-rater agreement Fleiss' kappa (κ), while the construct validity was evaluated with confirmatory factor analysis (CFA). Pearson's *r* and *t*-tests were used to examine, respectively, the structure, convergent, and divergent validity of the test and theory-consistent group differences. In addition, the test-retest (r_tt_) correlations were used to test the stability of the ADQ.

## Results

### Items and scales statistics

Table 2 summarizes the means, standard deviations, and Cronbach's α coefficients of the total sample. It shows that the internal consistencies of each scale of the ADQ are very high, with Cronbach's α coefficients ranging from 0.92 (for the ADQ-VD scale) to 0.96 (for the ADQ-ApA scale and the ADQ-AD scale). Excepting the AVA subscale of the ADQ-ArA, the αs are also high for the subscales of each ADQ scale (ranges from the 0.93 for the EMD subscale of the ADQ-AD to 0.73 for the AC subscale of the ADQ-AD). Apparently, the lowest numbers of items within subscales can explain the lowest α's (cf. the AVA from the ADQ-ArA or the AC from the ADQ-AD). According to the Spearman-Brown formula, these scales would achieve reliability of around 0.80 with 13 (instead of 5) and 12 (instead of 8) items, respectively.

**Table 2:** Means (*M*), standard deviations (*SD*), and Cronbach's α coefficients (on the total sample) of the four scales of the ADQ and their subscales.

**Scales of the ADQ**	***M***	***SD***	**Cronbach's α**
ADQ-ArA (*N* = 1,562)	14.52	10.47	0.94
SR	7.96	6.10	0.91
PP	4.42	3.72	0.85
AVA	2.14	1.50	0.61
ADQ-ApA (*N* = 1,499)	23.08	13.62	0.96
WT	6.58	4.28	0.89
AC	11.57	6.59	0.91
SR	4.94	3.52	0.86
ADQ-VD (*N* = 1,498)	10.58	8.29	0.92
NA	6.94	5.57	0.90
AA	3.64	3.12	0.84
ADQ-AD (*N* = 1,497)	18.30	15.86	0.96
EMD	8.30	7.88	0.93
PA	3.95	2.73	0.88
NA	3.76	2.34	0.85
AC	2.32	1.15	0.73

*ADQ-ArA, Anxiety and Depression Questionnaire—Arousal Anxiety; SR, Somatic Reactivity; PP, Panic/Phobia; AVA, Attentional Vigilance/Avoidance; ADQ-ApA, Anxiety and Depression Questionnaire—Apprehension Anxiety; WT, Worrisome Thoughts; AC, Attentional Control; SR, Somatic Reactivity; ADQ-VD, Anxiety and Depression Questionnaire—Valence Depression; NA, Negative Affect; AA, Attentional Avoidance; ADQ-AD, Anxiety and Depression Questionnaire—Anhedonic Depression; MD, Motivational Deficit; PA, Positive Affect; NA, Negative Affect; AC, Attentional Control*.

Table 3 demonstrates the means, standard deviations, as well as *t*-test results between the sexes, separately evaluated for each scale of the ADQ. It informs that women scored significantly higher on both types of anxiety. There were no significant sex differences on valence and anhedonic depression.

**Table 3:** Means (*M*), standard deviations (*SD*), and *t*-test comparisons between men and women of the four scales of the ADQ and their subscales.

**Scales of the ADQ**	***M***	***SD***	***M***	***SD***	***t***	***d***
	Men (*N* = 748)		Women (*N* = 814)			
ADQ-ArA	12.07	9.65	16.77	10.70	9.08^***^	0.46
SR	6.33	5.57	9.45	6.20	10.41^***^	0.53
PP	3.80	3.49	5.00	3.84	6.46^***^	0.33
AVA	1.94	1.43	2.32	1.53	5.01^***^	0.26
	Men (*N* = 711)		Women (*N* = 788)			
ADQ-ApA	20.46	13.07	25.45	13.67	7.21^***^	0.37
WT	5.94	4.28	7.17	4.48	5.42^***^	0.28
AC	10.43	6.46	12.59	6.54	6.39^***^	0.33
SR	4.09	3.26	5.70	3.57	9.08^***^	0.47
	Men (*N* = 707)		Women (*N* = 791)			
ADQ-VD	10.74	8.63	10.44	7.97	*n.s*.	
NA	6.59	5.57	7.26	5.56	2.30^*^	0.12
AA	4.15	3.73	3.19	2.23	5.32^***^	0.31
	Men (*N* = 707)		Women (*N* = 790)			
ADQ-AD	18.19	16.12	18.19	16.12	*n.s*.	
EMD	8.44	7.94	8.11	7.79	*n.s*.	
PA	3.99	3.65	3.91	2.18	n.s.	
NA	3.69	3.49	4.00	3.86	2.62^**^	0.08
AC	2.48	2.19	2.17	2.08	2.77^**^	0.15

ADQ-ArA, Anxiety and Depression Questionnaire-Arousal Anxiety; SR, Somatic Reactivity; PP, Panic/Phobia; AVA, Attentional Vigilance/Avoidance; ADQ-ApA, Anxiety and Depression Questionnaire-Apprehension Anxiety; WT, Worrisome Thoughts; AC, Attentional Control; SR, Somatic Reactivity; ADQ-VD, Anxiety and Depression Questionnaire-Valence Depression; NA, Negative Affect; AA, Attentional Avoidance; ADQ-AD, Anxiety and Depression Questionnaire-Anhedonic Depression; EMD, Emotional-Motivational Deficits; PA, Positive Affect; NA, Negative Affect; AC, Attentional Control;

* *p < 0.05*,

** *p < 0.01*,

*** *p < 0.001, n.s. non-significant*.

We extracted the “pure types” by controlling the level of the other three affective types. For example, we identified arousal anxiety when the individuals scored above the median in ADQ-ArA and below the median in the other three types (ADQ-ApA, ADQ-VD, ADQ-AD). Mixed types, on the other hand, were built of individuals who scored above the median on both types of anxiety (ADQ-ArA, ADQ-ApA) or depression (ADQ-VD, ADQ-AD). Interestingly, as Table 4 indicates, men reported all types of depression more frequently than women, while women declared arousal and mixed types of anxiety more frequently than men.

**Table 4:** The prevalence of “pure” affective types in women and men.

**Affective Type**	***N***	**Age**	**Sex**	
			**Men**	**Women**
Arousal type of anxiety	36	*M* = 44.03 *SD* = 13.13	13 (36.1%)	23 (63.9%)
Apprehension type of anxiety	27	*M* = 41.48 *SD* = 9.53	15 (55.6%)	12 (44.4%)
Mixed type of anxiety	43	*M* = 39.61 *SD* = 13.44	12 (27.9%)	31 (72.1%)
Valence type of depression	37	*M* = 38.78 *SD* = 14.11	24 (64.9%)	13 (35.1%)
Anhedonic type of depression	41	*M* = 39.39 *SD* = 12.84	24 (58.5%)	17 (41.5%)
Mixed type of depression	46	*M* = 35.09 *SD* = 11.07	30 (65.2%)	16 (34.8%)

*M, mean, SD, standard deviation*.

The discriminatory power coefficients of items from the four ADQ scales are reported in Table 5. Similarly to the construction stage, we calculated the Youle's Phi coefficients and proposed the value ≥0.30 as indicative of good and very good discrimination.

**Table 5:** Discriminatory power of items (Yule ϕ, phi-coefficient; on total sample) measuring arousal anxiety (ADQ-ArA), apprehension anxiety (ADQ-ApA), valence depression (ADQ-VD), and anhedonic depression (ADQ-AD).

**ANXIETY SCALES**											
**Subscales of ADQ-ArA**						**Subscales of ADQ-ApA**					
**SR**		**PP**		**AVA**		**WT**		**AC**		**SR**	
**Item**	**Yule's** ***Phi***	**Item**	**Yule's** ***Phi***	**Item**	**Yule's** ***Phi***	**Item**	**Yule's** ***Phi***	**Item**	**Yule's** ***Phi***	**Item**	**Yule's** ***Phi***
1	0.34	2	0.40	8	0.37	2	0.66	1	0.56	3	0.51
3	0.49	5	0.52	13	0.33	6	0.44	4	0.58	7	0.53
7	0.47	6	0.59	25	0.42	9	0.65	5	0.48	10	0.56
10	0.52	9	0.51	30	0.43	14	0.62	8	0.66	13	0.58
11	0.35	12	0.52	38	0.35	16	0.67	11	0.39	20	0.45
14	0.63	15	0.55			18	0.66	12	0.47	29	0.64
18	0.62	16	0.60			23	0.56	15	0.60	33	0.59
19	0.64	20	0.37			26	0.69	17	0.65	35	0.61
22	0.56	24	0.47			32	0.53	19	0.62	39	0.49
23	0.56	29	0.36			37	0.37	21	0.56	43	0.57
26	0.34	33	0.51			41	0.59	22	0.63	46	0.57
27	0.49	37	0.55			42	0.61	24	0.66		
28	0.58	40	0.57			44	0.60	25	0.43		
31	0.63	44	0.55			47	0.41	27	0.39		
32	0.68							28	0.60		
35	0.52							30	0.60		
36	0.55							31	0.55		
39	0.61							34	0.56		
41	0.57							36	0.44		
42	0.66							38	0.30		
43	0.59							40	0.43		
45	0.60							45	0.55		
								48	0.68		
**DEPRESSION SCALES**											
**Subscales of ADQ-VD**						**Subscales of ADQ-AD**					
**NA**		**AA**		**EMD**		**PA**		**NA**		**AC**	
**Item**	**Yule's** ***Phi***	**Item**	**Yule's** ***Phi***	**Item**	**Yule's** ***Phi***	**Item**	**Yule's** ***Phi***	**Item**	**Yule's** ***Phi***	**Item**	**Yule's** ***Phi***
3	0.56	2	0.42	2	0.62	1	0.53	4	0.65	3	0.52
4	0.37	5	0.39	6	0.46	5	0.54	9	0.62	8	0.47
6	0.60	8	0.44	7	0.41	10	0.41	13	0.65	15	0.44
7	0.51	10	0.52	11	0.49	14	0.44	17	0.61	22	0.43
9	0.63	15	0.44	12	0.56	18	0.55	20	0.67	29	0.36
11	0.37	17	0.49	16	0.51	21	0.50	25	0.69	33	0.38
12	0.46	19	0.50	19	0.53	26	0.68	30	0.58	48	0.43
14	0.53	22	0.46	23	0.54	28	0.59	36	0.51	55	0.36
16	0.48	24	0.51	24	0.58	32	0.55	39	0.51		
18	0.55	26	0.45	27	0.45	37	0.46	44	0.57		
20	0.52	28	0.47	31	0.50	40	0.67	49	0.60		
21	0.62	30	0.46	34	0.39	45	0.67	56	0.63		
23	0.55	34	0.49	35	0.56	50	0.64				
25	0.47	36	0.46	38	0.67						
27	0.50	40	0.42	41	0.62						
29	0.50			42	0.34						
31	0.52			43	0.46						
33	0.48			46	0.52						
35	0.63			47	0.44						
38	0.49			51	0.45						
39	0.43			52	0.58						
				53	0.60						
				54	0.63						
				57	0.68						
				58	0.57						
				59	0.51						
				60	0.57						
				61	0.48						
				62	0.65						
				63	0.60						
				64	0.62						

*Anxiety scales: SR, Somatic Reactivity; PP, Panic/Phobia; AVA, Attentional Vigilance/Avoidance; WT, Worrisome Thoughts; AC, Attentional Control; SR, Somatic Reactivity. Depression scales: NA, Negative Affect; AA, Attentional Avoidance; EMD, Emotional-Motivational Deficit; PA, Positive Affect; NA, Negative Affect; AC, Attentional Control*.

The results suggest high item discrimination coefficients, ranging from 0.33 (the AVA subscale) to 0.68 (the SR subscale) in the ADQ-ArA, from 0.30 (the AC subscale) to 0.69 (the WT subscale) in the ADQ-ApA, from 0.37 (the NA subscale) to 0.63 (the NA subscale) in the ADQ-VD, and from 0.34 (the EMD subscale) to 0.69 (the NA subscale) in the ADQ-AD.

In order to check the obtained intercorrelations among subscales composing adequate scales of the ADQ in study 1, we analyzed them in study 2. Table 6 demonstrates that generally all of the results are confirmed. However, comparing these findings to the findings from study 1, all correlation coefficients, across all scales of the questionnaire, increased.

**Table 6:** Intercorrelations among subscales in the four scales of the ADQ.

**Subscales of ADQ-ArA**	**SR**	**PP**	**AVA**	
SR		0.42^***^	0.43^***^	
PP	0.42^***^		0.46^***^	
AVA	0.43^***^	0.46^***^		
**Subscales of ADQ-ApA**	**WT**	**AC**	**SR**	
WT		−0.57^***^	0.53^***^	
AC	−0.57^***^		−0.46^***^	
SR	0.53^***^	−0.46^***^		
**Subscales of ADQ-VD**	**NA**	**AA**		
NA		0.64^***^		
AA	0.64^***^			
**Subscales of ADQ-AD**	**EMD**	**PA**	**NA**	**AC**
EMD		−0.82^***^	0.84^***^	−0.69^***^
PA	−0.82^***^		−0.73^***^	0.48^***^
NA	0.84^***^	−0.73^***^		−0.63^***^
AC	−0.69^***^	0.48^***^	−0.63^***^	

*ADQ-ArA, Anxiety and Depression Questionnaire—Arousal Anxiety; SR, Somatic Reactivity; PP, Panic/Phobia; AVA, Attentional Vigilance/Avoidance; ADQ-ApA, Anxiety and Depression Questionnaire—Apprehension Anxiety; WT, Worrisome Thoughts; AC, Attentional Control; SR, Somatic Reactivity; ADQ-VD, Anxiety and Depression Questionnaire—Valence Depression; NA, Negative Affect; AA, Attentional Avoidance; ADQ-AD, Anxiety and Depression Questionnaire—Anhedonic Depression; EMD, Emotional-Motivational Deficit; PA, Positive Affect; NA, Negative Affect; AC, Attentional Control; Pearson's r*,

*** *p < 0.001*.

### Validity of the ADQ

In the case of *content validity*, we examined the extent to which the particular scales of the ADQ represent all proposed facets of arousal anxiety (ADQ-ArA), apprehension anxiety (ADQ-ApA), valence depression (ADQ-VD), and anhedonic depression (ADQ-AD) constructs. Thus, three experts evaluated whether items of the ADQ-ArA and ADQ-AD assess the defined content of arousal anxiety and anhedonic depression, and another three experts decided if the test positions of the ADQ-ApA and ADQ-VD cover the content of apprehension anxiety and valence depression, respectively. Precisely, the raters were instructed to assign the items measuring the adequate construct (e.g., arousal anxiety) to the distinguished facets of that construct (e.g., SR, PP, AVA). The inter-rater agreement was measured with Fleiss' kappa (Fleiss, 1971) using an online calculator (Geertzen, 2012). Kappa (κ) ranges from 0 to 1, with higher values showing greater inter-rater reliability of agreement. For all tested versions of the ADQ the κ-values were very high: ADQ-ArA, κ = 0.93; ADQ- ApA, κ = 0.90; ADQ-VD, κ = 0.94; ADQ-AD, κ = 0.98. Then the raters' sorting was compared to the scoring keys in order to replace, remove or linguistically correct problematic items.

The CFA was used to evaluate the *factorial validity* and authorize the results from Study 1. Along with these findings we tested (Mplus; Muthén and Muthén, 1998) the same models; however, they were formed on the corrected versions of the ADQ elaborated according to the results from Study 1 (e.g., they have a different number of items because some of them were removed from the original versions, different number of subscales). Again, χ^2^ values for the models fit across all version of the ADQ were significant, thus other fit indices were reported (Kline, 2005).

The results of the CFA for all scales of ADQ are presented in Tables 7, 8. As can be seen, the three-factor model showed the best fit in case of the ADQ-ArA with all indices reaching satisfactory levels. The data clearly demonstrated that all the items are proper markers of the expected single factor. More precisely, for the SR factor range from 0.42 to 0.87, for the PP factor from 0.54 to 0.82, and for the AVA from 0.49 to 0.74.

**Table 7:** Goodness of fit indices for the two models of Anxiety and Depression Questionnaire—Arousal Anxiety (ADQ-ArA); for the two models of Anxiety and Depression Questionnaire—Apprehension Anxiety (ADQ-ApA); for the two models of Anxiety and Depression Questionnaire—Valence Depression (ADQ-VD), and for the three models of Anxiety and Depression Questionnaire—Anhedonic Depression (ADQ-AD).

**Goodness of fit indices**	**One-factor model**	**Three-factor model**	
**ADQ-ArA**			
RMSEA	0.054	0.053	
CFI	0.92	0.94	
TLI	0.92	0.93	
*χ^2^*/df	5.40	5.31	
**ADQ-ApA**			
RMSEA	0.060	0.058	
CFI	0.91	0.92	
TLI	0.90	0.91	
*χ^2^*/df	6.44	6.11	
**ADQ-VD**			
**Goodness of fit indices**	**One-factor model**	**Two-factor model**	
RMSEA	0.061	0.053	
CFI	0.89	0.92	
TLI	0.89	0.92	
*χ^2^*/df	6.66	5.17	
**ADQ-AD**			
**Goodness of fit indices**	**One-factor model**	**Three-factor model**	**Four-factor model**
RMSEA	0.044	0.043	0.041
CFI	0.93	0.93	0.94
TLI	0.93	0.94	0.94
*χ^2^*/df	3.89	3.72	3.56

**Table 8:** Factor loadings of items for the three-factor model of Anxiety and Depression Questionnaire—Arousal Anxiety (ADQ-ArA), for the three-factor model of Anxiety and Depression Questionnaire—Apprehension Anxiety (ADQ-ApA), for the two-factor model of Anxiety and Depression Questionnaire—Valence Depression (ADQ-VD), and for the four-factor model of Anxiety and Depression Questionnaire—Anhedonic Depression (ADQ-AD).

**ADQ-ArA**						**ADQ-ApA**						**ADQ-VD**						**ADQ-AD**					
**SR**		**PP**		**NA**		**EMD**		**PA**		**NA**		**AC**		**AA**		**EMD**		**PA**		**NA**		**AC**	
**Item**	**Factor loading**	**Item**	**Factor loading**	**Item**	**Factor loading**	**Item**	**Factor loading**	**Item**	**Factor loading**	**Item**	**Factor loading**	**Item**	**Factor loading**	**Item**	**Factor loading**	**Item**	**Factor loading**	**Item**	**Factor loading**	**Item**	**Factor loading**	**Item**	**Factor loading**
1	0.45	2	0.54	8	0.74	2	0.83	1	0.72	3	0.71	3	0.71	2	0.55	2	0.79	1	0.79	4	0.82	3	0.72
3	0.64	5	0.70	13	0.49	6	0.58	4	0.77	7	0.72	4	0.58	5	0.56	6	0.70	5	0.75	9	0.82	8	0.70
7	0.64	6	0.80	25	0.63	9	0.81	5	0.62	10	0.75	6	0.76	8	0.61	7	0.56	10	0.59	13	0.86	15	0.88
10	0.67	9	0.66	30	0.55	14	0.82	8	0.84	13	0.78	7	0.67	10	0.71	11	0.65	14	0.67	17	0.83	22	0.52
11	0.47	12	0.75	38	0.69	16	0.86	11	0.52	20	0.61	9	0.79	15	0.64	12	0.73	18	0.77	20	0.86	29	0.49
14	0.80	15	0.71			18	0.84	12	0.64	29	0.84	11	0.53	17	0.69	16	0.69	21	0.67	25	0.88	33	0.55
18	0.78	16	0.77			23	0.72	15	0.76	33	0.78	12	0.66	19	0.81	19	0.68	26	0.85	30	0.78	48	0.73
19	0.82	20	0.50			26	0.87	17	0.82	35	0.83	14	0.75	22	0.70	23	0.74	28	0.80	36	0.76	55	0.45
22	0.74	24	0.66			32	0.68	19	0.79	39	0.68	16	0.58	24	0.68	24	0.76	32	0.74	39	0.66		
23	0.73	29	0.47			37	0.51	21	0.75	43	0.77	18	0.74	26	0.68	27	0.59	37	0.72	44	0.70		
26	0.42	33	0.82			41	0.77	22	0.80	46	0.78	20	0.75	28	0.74	31	0.68	40	0.86	49	0.80		
27	0.74	37	0.81			42	0.77	24	0.73			21	0.81	30	0.66	34	0.59	45	0.85	56	0.81		
28	0.79	40	0.81			44	0.78	25	0.57			23	0.74	34	0.69	35	0.74	50	0.82				
31	0.69	44	0.81			47	0.54	27	0.52			25	0.60	36	0.66	38	0.83						
32	0.87							28	0.77			27	0.76	40	0.58	41	0.78						
35	0.69							30	0.78			29	0.73			42	0.51						
36	0.72							31	0.70			31	0.77			43	0.78						
39	0.78							34	0.74			33	0.68			46	0.67						
41	0.67							36	0.59			35	0.80			47	0.60						
42	0.84							38	0.41			38	0.64			51	0.64						
43	0.76							40	0.59			39	0.58			52	0.77						
45	0.77							45	0.74							53	0.81						
								48	0.85							54	0.82						
																57	0.85						
																58	0.73						
																59	0.67						
																60	0.76						
																61	0.67						
																62	0.84						
																63	0.79						
																64	0.79						

*ADQ-ArA: SR, Somatic Reactivity; PP, Panic/Phobia; AVA, Attentional Vigilance/Avoidance; ADQ-ApA: WT, Worrisome Thoughts; AC, Attentional Control; SR, Somatic Reactivity; ADQ-VD: NA, Negative Affect; AA, Attentional Avoidance; ADQ-AD: EMD, Emotional- Motivational Deficit; PA, Positive Affect; NA, Negative Affect; AC, Attentional Control; loadings below 0.40 are omitted*.

In case of the ADQ-ApA, the three-factor model had better fit parameters than the one-factor model. The factors loadings for items of the three-factor model are high: from 0.51 to 0.87 for the WT subscale, from 0.41 to 0.85 for the AC subscale, and from 0.61 to 0.84 for the SR subscale.

Again, the two-factor model for the ADQ-VD seemed to be a better fit than the one-factor solution, and all of the items loaded high on the adequate factor: for NA from 0.53 to 0.80, and for AA from 0.55 to 0.81.

The best solution for the ADQ-AD is the four-factor model and the factor loadings of items for this model reach a satisfactory level. More specifically, for the EMD subscale from 0.51 to 0.85, for the PA subscale from 0.59 to 0.86, for the NA subscale from 0.70 to 0.88, and for the AC subscale from 0.52 to 0.88.

Additionally, in order to place the proposed affective types among other related personality constructs, we assessed *the convergent and divergent validity* with well-recognized measures. We predicted that state-like arousal anxiety and trait-like apprehension anxiety are both positively related to state anxiety and trait anxiety (STAI). However, the correlation between state anxiety and arousal anxiety should be higher than the correlation between trait anxiety and arousal anxiety, and opposite relations should be identified for apprehension anxiety. As can be seen from Table 9, the obtained data confirmed these predictions.

**Table 9:** Correlations between arousal anxiety (ADQ-ArA) and state and trait anxiety (STAI), extraversion and neuroticism (EPQ-R [S]), positive and negative affect (PANAS-X; instruction “always”), and correlations between apprehension anxiety (ADQ-ApA) and state and trait anxiety (STAI), extraversion and neuroticism (EPQ-R [S]), positive and negative affect (PANAS-X; instruction “always”), and attentional control (ACS).

	**State anxiety (*N* = 1,506)**	**Trait anxiety (*N* = 1,466)**	**Extraversion (*N* = 1,470)**	**Neuroticism (*N* = 1,470)**	**PA (*N* = 1,470)**	**NA (*N* = 1,470)**	
ADQ-ArA	0.68^***^	0.52^***^	−0.38^***^	0.69^***^	−0.40^***^	0.59^***^	
	**State anxiety (*N* = 1,469)**	**Trait anxiety (*N* = 1,417)**	**Extraversion (*N* = 1,470)**	**Neuroticism (*N* = 1,470)**	**PA (*N* = 1,448)**	**NA (*N* = 1,448)**	**Attentional control (*N* = 1,412)**
ADQ-ApA	0.59^***^	0.62^***^	−0.39^***^	0.79^***^	−0.43^***^	0.62^***^	−0.62^***^

*Pearson's r*,

*** *p < 0.001; PA, Positive Affect; NA, Negative Affect*.

The review of results from other sources showed that a moderate negative correlation with extraversion and a moderate and high positive correlation with neuroticism (EPQ-R[S]) is usually obtained for both state and trait anxiety (STAI) (see Fajkowska, 2013). Thus, similar relations between extraversion, neuroticism, arousal anxiety, and apprehension anxiety could be expected, which is actually reflected in the results showed in Table 9.

According to the tripartite model of anxiety and depression proposed by Clark and Watson (Burns and Eidelson, 1998; Watson, 2000), anxiety relates to Negative Affect (NA) but is not connected with Positive Affect (PA), while depression is associated with both affects by correlating negatively with PA and positively with NA. The results of our studies only partially support this model. Both types of anxiety moderately and positively related to NA; however, they also correlated moderately and negatively with PA (see Table 9). But both types of depression, low and negatively (valence depression) and moderately and negatively (anhedonic depression) related to PA, and moderately and positively to NA (see **Table 11**). Nonetheless, there are some studies matching our (but not Clark and Watson's) findings (e.g., Burns and Eidelson, 1998; Fajkowska and Marszał-Wiśniewska, 2009).

Fajkowska and Derryberry (2010) provided evidence that anxious and depressive subjects scored significantly lower on effortful attentional control than non-anxious and non-depressive individuals. The results from our study are congruent with their findings. As Tables 9, 10 show, apprehension anxiety and anhedonic depression are negatively correlated with attentional control.

**Table 10:** Correlations between valence depression (ADQ-VD) and depressive tendencies (BDI); extraversion and neuroticism (EPQ-R [S]) and correlations between anhedonic depression (ADQ-AD) and depressive tendencies (BDI), extraversion and neuroticism (EPQ-R [S]), and attentional control (ACS).

	**Depressive tendencies (*N* = 1,498)**	**Extraversion (*N* = 1,424)**	**Neuroticism (*N* = 1,424)**	
**ADQ-VD**	0.36^***^	−0.36^***^	0.50^***^	
	**Depressive tendencies (*N* = 1,497)**	**Extraversion (*N* = 1,428)**	**Neuroticism (*N* = 1,428)**	**Attentional control (*N* = 1,385)**
**ADQ-AD**	0.60^***^	−0.44^***^	0.51^***^	−0.43^***^

*Pearson's r*,

*** *p < 0.001*.

The Mood and Anxiety Symptom Questionnaire (MASQ; Watson, 2000) can be used to assess anhedonic depression. The MASQ Anhedonic Depression Subscale displays good convergent validity with the Beck Depression Inventory (BDI; cf. Kendall et al., 1987). Therefore, we predicted a higher and positive correlation between anhedonic depression and depression measured by BDI, and low or moderate between valence depression and depression measured by BDI. This forecast is supported by the data presented in Table 10.

Generally, in most studies low extraversion and high neuroticism are found in clinical and nonclinical depression (e.g., Watson et al., 1999; Kotov et al., 2010; Fajkowska, 2013). Thus, it implies that we should expect that both types of depression would be negatively related to extraversion and positively to neuroticism. Indeed, the data presented in Table 10 support this hypothesis.

The structure of both types of depression refers to the NA; however, its content is depression-type specific (cf. definition of valence and anhedonic depression). We predicted that valence depression should correlate higher with hostility than anhedonic depression, while anhedonic depression would be more strongly related to sadness and guilt. Fear should not differentiate depressions. The obtained data supported these speculations (cf. Table 11).

**Table 11:** Correlations between valence depression (ADQ-VD; *N* = 1,424) and anhedonic depression (ADQ-AD; *N* = 1,428) with PANAS-X (instruction “always”).

**PANAS-X scales**	**Valence depression**	**Anhedonic depression**
Positive affect	−0.21^***^	−0.40^***^
Negative affect	0.53^***^	0.55^***^
**BASIC NEGATIVE EMOTION SCALES**		
Fear	0.52^***^	0.54^***^
Sadness	0.48^***^	0.56^***^
Guilt	0.50^***^	0.56^***^
Hostility	0.57^***^	0.45^***^
**BASIC POSITIVE EMOTION SCALES**		
Joviality	−0.35^***^	−0.47^***^
Self-assurance	−0.31^***^	−0.41^***^
Attentiveness	−0.30^***^	−0.36^***^
**OTHER AFFECTIVE STATES**		
Shyness	0.47^***^	0.52^***^
Fatigue	0.40^***^	0.47^***^
Serenity	−0.23^***^	−0.31^***^
Surprise	0.26^***^	0.22^***^

*Pearson's r*,

*** *p < 0.001*.

In addition, the very low PA is a part of the structure of anhedonic depression, thus we expected stronger negative relations between anhedonic depression and the Basic Positive Scales (Joviality, Self-assurance, and Attentiveness) than between valence depression and these scales. Again, these expectations are reflected in our empirical data (cf. Table 11).

Finally, as the emotional-motivational deficit defines anhedonic depression we also predicted stronger relations between Other Affective States, especially those referring to low energetic states (Fatigue, Serenity), and anhedonic depression than between Other Affective States and valence depression. As Table 11 shows, the predictions were confirmed.

We assumed that reactive types, that is arousal anxiety (ArA) and valence depression (VD), should be more weakly associated with adaptive and nonadaptive cognitive strategies of emotion regulation than regulative types, that is apprehension anxiety (ApA) and anhedonic depression (AD). Data presented in Table 12 qualified our predictions.

**Table 12:** Correlations between reactive types—arousal anxiety (ADQ-ArA) and valence depression (ADQ-VD), regulative types—apprehension anxiety (ADQ-ApA), anhedonic depression (ADQ-AD) and adaptive and nonadaptive strategies of emotion regulation (CERQ).

	**Adaptive strategies**	**Nonadaptive strategies**
**REACTIVE TYPES**		
ADQ-ArA (*N* = 1,401)	−0.13^**^	0.18^**^
ADQ-VD (*N* = 1,480)	−0.23^**^	0.32^**^
**REGULATIVE TYPES**		
ADQ-ApA (*N* = 1,401)	−0.28^**^	0.45^**^
ADQ-AD (*N* = 1,480)	−0.41^**^	0.52^**^

*Pearson's r*,

** *p < 0.01*.

The next step in assessing construct validity was to analyze the *theory-consistent group differences*. Gray (1981) proposed two systems of controlling behavioral activity, that is the behavioral inhibition system (BIS) and the behavioral activation system (BAS). The BIS is thought to regulate aversive motives, in which the goal is to move away from something unpleasant, while the BAS is understood to regulate appetitive motives, in which the goal is to move toward something desirable (Carver and White, 1994).

It claims that the amygdala provides inputs to the BIS and may relay its outputs to the hypothalamus and autonomic nervous system, thereby mediating anxious arousal. Sustained activation of the BIS may therefore account for some features of anxiety and be related to panic (cf. Barlow, 2004, p. 210). Thus, we assumed that high arousal-anxious individuals would score higher on the BIS than low arousal-anxious ones. And it is clear from the results that high arousal-anxious subjects (*n* = 265) are higher (*M* = 3.05, *SD* = 0.47) on the BIS (Carver and White, 1994; Müller and Wytykowska, 2005) scale than low arousal-anxious participants (*n* = 313; *M* = 2.50, *SD* = 0.47), *t*_(576)_ = 14.00, *p* < 0.001, *d* = 1.18. However, as data from one study (Moser et al., 2013) indicated, we should expect higher BIS in apprehension anxiety (measured by STAI) than in arousal anxiety (measured by MASQ). Their study showed that apprehension anxiety correlates three times higher with BIS than arousal anxiety because the former one is most closely associated with error monitoring. The findings from our study employing ADQ-ArA and ADQ-ApA measures are in accord with the cited studies: high apprehension-anxious individuals (*n* = 134) are higher (*M* = 2.88, *SD* = 0.39) on the BIS scale than high arousal-anxious participants (*n* = 133; *M* = 2.72, *SD* = 0.44), *t*_(265)_ = 3.49, *p* < 0.001, *d* = 0.44. The high-apprehension subjects (*n* = 298) obtained higher scores on BIS than low-apprehension individuals (*n* = 301; *M* = 3.12, *SD* = 0.46, and *M* = 2.47, *SD* = 0.37, respectively; *t*_(597)_ = 17.16, *p* < 0.001, *d* = 1.40).

In addition, high activity of the BIS means a higher level of sensitivity to nonreward, punishment, and novel experience, which results in a natural avoidance of such environments in order to prevent negative experiences such as fear, anxiety, frustration, and sadness. Thus, it should be predicted that individuals with a high level of valence depression would show a higher level of BIS comparing to individuals with a low level of valence depression and high-anhedonic depressive, because the negative affect building this type of depression relates to the aforementioned range of negative emotions. Indeed, results of the analysis aimed at these differences showed that individuals with high valence depression (*n* = 296), measured with ADQ-VD, revealed a higher BIS level (*M* = 2.92, *SD* = 0.43) than subjects with low valence depression (*n* = 319; *M* = 2.51, *SD* = 0.42), *t*_(613)_ = 10.84, *p* < 0.001, *d* = 0.87, and participants with high valence depression (*n* = 122) scored higher on the BIS scale than those with high anhedonic depression as assessed by the ADQ-AD (*n* = 124; *M* = 2.87, *SD* = 0.40, and *M* = 2.75, *SD* = 0.47, respectively), *t*_(244)_ = 2.13, *p* < 0.05, *d* = 0.30).

Bijttebier et al. (2009) summarized the studies that examined the relationship between sensitivity of the BIS and BAS systems and a broad range of psychiatric disorders. Among others, they found that low BAS sensitivity characterized anhedonic depression. Along with these results we assessed the differences in BAS level and three BAS-related scales: BAS Drive, BAS Fun Seeking, and BAS Reward Responsiveness (Carver and White, 1994; Müller and Wytykowska, 2005) between high anhedonic-depressive (*n* = 263) and low anhedonic-depressive (*n* = 300) individuals, and between high anhedonic-depressive (*n* = 124) and high valence-depressive (*n* = 122). Anhedonic depression and valence depression were assessed by the ADQ-VD and ADQ-AD, respectively. As expected, the results showed that high anhedonic-depressive individuals scored significantly lower on the BAS (*M* = 2.52, *SD* = 0.50) than low anhedonic-depressive (*M* = 2.86, *SD* = 0.43), *t*_(561)_ = 8.54, *p* < 0.001, *d* = 0.72, and high valence-depressive individuals (*M* = 2.54, *SD* = 0.48, and *M* = 2.77, *SD* = 0.43, respectively), *t*_(244)_ = 3.89, *p* < 0.001, *d* = 0.50. The high anhedonic-depressive participants were significantly lower (*M* = 2.85, *SD* = 0.54) on the BAS Drive scale than low anhedonic-depressive participants (*M* = 5.41, *SD* = 1.82), *t*_(561)_ = 7.87, *p* < 0.001, *d* = 0.68, and they were lower on the BAS Fun Seeking (*M* = 2.58, *SD* = 0.55) and BAS Reward Responsiveness (*M* = 2.98, *SD* = 0.51) scales than low anhedonic-depressive individuals (*M* = 2.88, *SD* = 0.45, *t*_(561)_ = 6.99, *p* < 0.001, *d* = 0.58 and *M* = 3.27, *SD* = 0.38, *t*_(561)_ = 7.76, *p* < 0.001, *d* = 0.66, respectively). Also, the high anhedonic-depressive participants were lower on BAS drive, BAS Fun Seeking, and BAS Reward Responsiveness scales than high valence-depressive individuals [*M* = 2.47, *SD* = 0.55 vs. *M* = 2.72, *SD* = 0.56, *t*_(244)_ = 3.54, *p* < 0.001, *d* = 0.45; *M* = 2.62, *SD* = 0.53 vs. *M* = 2.82, *SD* = 0.49, *t*_(244)_ = 3.21, *p* < 0.001, *d* = 0.39; *M* = 3.03, *SD* = 0.45 vs. *M* = 3.20, *SD* = 0.46, *t*_(244)_ = 2.86, *p* < 0.01, *d* = 0.37, respectively).

### Estimation of the questionnaire test-retest reliability

The test-retest (r_tt_) reliabilities were evaluated on smaller groups (randomly selected from the total sample) that filled out the ADQ scales again after 5 weeks from the initial study. According to the results, the r_tt_ reliabilities (see Table 13) are high for all scales of the ADQ, varying from 0.70 (ADQ-ArA) to 0.89 (ADQ-ApA). Moreover, coefficients for the tests measuring state-like arousal anxiety (ADQ-ArA) and valence depression (ADQ-VD) are lower (0.70, 0.79, respectively) than for the tests assessing trait-like apprehension anxiety (ADQ-ApA) and anhedonic depression (APQ-AD); 0.89 and 0.88, respectively. Coefficients of 0.70 are considered satisfactory for personality states (Spielberger, 1983). Additionally, the test-retest reliability of most subscales of each ADQ scale is high or moderate, except for poor reliability (0.45) of the subscale of the ADQ-ArA version that assesses attentional vigilance/avoidance (AVA).

**Table 13:** Test-retest (r_tt_) reliabilities of the four scales of ADQ and their subscales (five week retest interval).

**ADQ**	**r_tt_**
ADQ-ArA (*N* = 139)	0.70^***^
SR	0.78^***^
PP	0.71^**^
AVA	0.45^**^
ADQ-ApA (*N* = 128)	0.89^***^
WT	0.73^***^
AC	0.77^**^
SR	0.65^**^
ADQ-VD (*N* = 145)	0.79^***^
NA	0.81^***^
AA	0.67^**^
ADQ-AD (*N* = 142)	0.88^***^
EMD	86^***^
PA	0.77^***^
NA	0.85^***^
AC	0.61^***^

*ADQ-ArA, Anxiety and Depression Questionnaire—Arousal Anxiety; SR, Somatic Reactivity; PP, Panic/Phobia; AVA, Attentional Vigilance/Avoidance; ADQ-ApA, Anxiety and Depression Questionnaire—Apprehension Anxiety; WT, Worrisome Thoughts; AC, Attentional Control; SR, Somatic Reactivity; ADQ-VD, Anxiety and Depression Questionnaire—Valence Depression; NA, Negative Affect; AA, Attentional Avoidance; ADQ-AD, Anxiety and Depression Questionnaire—Anhedonic Depression; EMD, Emotional-Motivational Deficits; PA, Positive Affect; NA, Negative Affect; AC, Attentional Control*;

*** *p < 0.001*,

** *p < 0.01, p < 0.05*.

## Discussion

The aim of the present studies was to validate a recently proposed typology of anxiety and depression operationalized within the systemic approach to personality trait and personality type (Fajkowska, 2013) and to develop a questionnaire based on it. This typology has been offered as a supplement to the widely accepted categorizations (e.g., Spielberger, 1983; Heller, 1993a,b; Watson, 2000; American Psychiatric Association, 2013) with the intention to advance knowledge in differential and overlapping features between anxiety and depression, and in differential and overlapping adaptive meanings of both phenomena, especially in non-clinical forms of anxiety/depression. In this approach, anxiety and depression are seen as complex personality types and their new grouping refers to their specific structural composition (mechanisms, components, and behavioral markers) and the dominant functions they play in stimulation processing (reactive, regulative). Hence, six affective types are proposed: arousal anxiety, apprehension anxiety, mixed anxiety, valence depression, anhedonic depression, and mixed depression. It is assumed that differences and similarities in structural components and dominant functions in stimulation processing in various affective types are connected with differences and similarities in their adaptive meanings. This line of reasoning suggests that one can expect more out-group than in-group similarities or more in-group than out-group differences. This theoretical proposition concerning a new typology of anxiety and depression has led us to develop a questionnaire that corresponds fully to this model. The empirical data gathered across the two stages—construction and validation—allowed us to offer the final form of the Anxiety and Depression Questionnaire (ADQ). The ADQ is composed of four multidimensional versions, directly assessing Arousal Type of anxiety (ADQ-ArA), Apprehension Type of anxiety (ADQ-ApA), Valence Type of depression (ADQ-VD), and Anhedonic Type of depression (ADQ-AD), and indirectly evaluating Mixed Type of anxiety (MA) and Mixed Type of depression (MD). The results showed that all scales of the ADQ are valid and reliable measurements.

The gender differences we found with the ADQ were to some extent similar to those found by other authors (see Fox, 2008 for a review). The prevalence of mood disorders is generally much higher among women than men. In our studies women scored higher on both types of anxiety, but we did not observe sex differences in valence and anhedonic depressions. However, when we used the “pure types” (where we controlled the level of the other three affective disorders) the results indicated that men showed all types of depression more frequently than women, while women more frequently reported arousal and mixed types of anxiety. The probable explanations include the sensitivity of the measurement we used, or it might be possible that these results reflect social and cultural changes predisposing men to be more vulnerable to mood disorders, especially to depression, than women. This issue needs further and cross-cultural studies.

All scales of the ADQ are characterized by high homogeneity (cf. discrimination coefficients and Cronbach's α coefficients). However, across two studies, the intercorrelations among subscales composing the anhedonic depression scale of ADQ seem to be much higher than expected. The scales with the highest intercorrelations were emotional-motivational deficits (EMD) with both affects, positive (PA; −0.65 in Study 1 and −0.82 in Study 2) and negative (NA; 0.71 in Study 1 and 0.84 in Study 2). It might suggest that motivational and affective systems are not separable or that the selected items in these subscales need further elaboration. Nonetheless, future validation studies should provide more information on how to deal with this puzzle.

The results of content validity supported the theoretical assumptions regarding the internal structure of the proposed affective types. In addition, the CFA sustained the adequacy of these theoretical assumptions.

Apart from that, all scales of the ADQ also have good convergent and divergent validity. It is shown by ADQ-ArA's higher correlation with STAI state anxiety than STAI trait anxiety, ADQ-ApA's higher correlations with STAI trait anxiety than STAI state anxiety, ADQ-VD's lower correlation with BDI and higher ADQ-AD's correlation with BDI, which measures anhedonia as is assumed. Moreover, as expected, all types of anxiety and depression related positively to neuroticism and negatively to extraversion (EPQ-R[S]).

Contrary to the predictions stemming from the tripartite model of anxiety and depression (Watson, 2000), both types of anxiety and both types of depression related negatively to PA and positively to NA (PANAS-X). Our results are in line with other studies (see Fajkowska and Marszał-Wiśniewska, 2009 for a review) showing that anxiety is related to positive affect. However, the expected correlational patterns were found for valence and anhedonic depression with respect to the proposed theoretical structure of these affective types. Valence depression correlated higher with hostility than anhedonic depression, and anhedonic depression was more strongly related to sadness and guilt than valence depression. Moreover, anhedonic depression showed higher negative correlations with the basic positive emotions, and stronger positive correlations with fatigue and serenity than valence depression (PANAS-X).

Referring to the identified dominant functions in controlling stimulation, reactive or regulative, in affective types, we discovered that reactive types (arousal anxiety and valence depression) are more weakly related to strategies of emotion regulation (CERQ) than regulative types (apprehension anxiety and anhedonic depression). These results also support theoretical assumptions regarding the fact that regulative personality traits or types can be recognized through their correspondence to different strategies.

The ADQ is also characterized by satisfactory construct validity as measured by means of the theory-consistent group differences. There were significant differences in BIS levels (BIS/BAS scales) among high arousal-anxious, low arousal-anxious, high apprehension-anxious, and low-apprehension anxious. BIS relates to arousal, panic, and also to monitoring errors. In line with the results obtained by other authors (e.g., Moser et al., 2013), a higher level of BIS is more typical for apprehension anxiety as it is more associated with error monitoring than arousal anxiety. Obviously, both types of anxiety revealed a higher BIS level than individuals low on both arousal and apprehension anxiety. In addition, as BIS goes with fear, anxiety, frustration, and sadness (Gray, 1981), it was not surprising that it was higher in valence depression than in anhedonic depression and in low valence depression.

Finally, significant differences were found for the level of BAS and BAS-related scales: BAS Drive, BAS Fun Seeking, and BAS Reward Responsiveness in scoring higher on anhedonic depression compared to low anhedonic-depressive and valence-depressive. It is explained by the fact that low BAS is associated with anhedonia, i.e., with difficulties in goal achievement, impossibility to experience pleasure, and failure in delivering sufficient reward following approach behaviors.

It should also be added that the stability of all scales of the ADQ as measured by the test-retest technique is satisfactory.

Certain limitations of our study should be noted. First relates to the self-reported data that could be associated with several potential sources of bias and requires replication and confirmation with experimental procedures. Second, these studies were time consuming and demanding for the participants. Thus, a possibility existed that they clicked through the questionnaires without much reflection (although it seemed that the number of such participants was not especially high, and we tried to exclude these cases from analyses). Third, in statistical techniques like Cronbach's α or factor analysis (CFA), high parameter values sometimes indicated redundancy in scales. Even though we were struggling for both (a) the content differentiation of the scales and subscales and (b) good psychometric parameters, it was not always possible to achieve. Fourth, we used a median split for grouping participants and that method has its serious limitations; thus for further analysis we recommend considering different approaches (e.g., means and standard deviations). Fifth, the number of items measuring attentional subscales is too low, although all definitional aspects of each attentional construct are covered. This is usually the problem when one operationalizes processual elements of personality (cf. Pavlovian Temperament Survey; Attentional Control Scale). Definitely it needs further elaboration. Finally, there are more processes involved in producing anxiety and depression than taken here into consideration. We are aware that this model is far from being complete but it suggests the right direction—understanding affective types as complex, three-level systems.

Despite these limitations, we received satisfactory empirical support for the proposed typology of anxiety and depression. From the viewpoint of developing a reliable and valid instrument for self-ratings of affective types, the results provide evidence to support the good psychometric status of the ADQ as a measure for evaluation of proposed types of anxiety and depression. It should be pointed out, however, that the research described in this paper is not intended to provide normative data. Although the present studies are based on large samples, we believe that the pattern of results needs further replications.

Future research should also consider the utility of the ADQ and the extent to which it may be generalized in a variety of applied settings, for example clinical, educational, and work settings. Such studies may potentially reveal interesting information regarding the usefulness of the ADQ in both research and practice.

## Author contributions

MF: Provided the theoretical framework and supervised the project; MF, ED, and AW: Designed the questionnaire and contributed to the study design; ED: Analyzed the data and supervised data collection; MF: Drafted the manuscript; ED and AW: Provided critical revisions.

### Conflict of interest statement

The authors declare that the research was conducted in the absence of any commercial or financial relationships that could be construed as a potential conflict of interest.
