# Supplementary material for: Types of Anxiety and Depression: Theoretical Assumptions and Development of the Anxiety and Depression Questionnaire
Source: Front Psychol. 2018 Jan 23;8:2376. doi: 10.3389/fpsyg.2017.02376 (PMC5787098; doi:10.3389/fpsyg.2017.02376)
Supplement: Supplementary file 1 [file DataSheet1.pdf]

Anxiety and Depression Questionnaire (ADQ-AD)

Małgorzata Fajkowska, Agata Wytykowska, Joanna Kantor-Martynuska
& Ewa Domaradzka

Name and surname/ or nickname ....
Date of birth ....Sex ....
Education ....E-mail address..

This questionnaire contains statements that concern various emotions, experiences, and behaviors. Each statement can be responded to by circling "Agree" or "Disagree". Please mark the answer that describes you better. Do not spend too much time on individual statements. There are no good or bad responses. Please respond to all of the statements provided below.

1.	I am usually in a good mood.	Agree/ Disagree
2.	I feel completely bored.	Agree/ Disagree
3.	It usually takes some time for me to realize that something nice is happening around me.	Agree/ Disagree
4.	I often feel sad.	Agree/ Disagree
5.	I feel confident.	Agree/ Disagree
6.	I feel lively and full of energy.	Agree/ Disagree
7.	I have lost all interest in sex.	Agree/ Disagree
8.	When someone tells a story that is full of various emotions, I feel that my thoughts drift away.	Agree/ Disagree
9.	I am disappointed with myself.	Agree/ Disagree
10.	I do not feel lonely.	Agree/ Disagree
11.	I cannot enjoy it when someone praises or expresses appreciation of me.	Agree/ Disagree

12.	I feel slowed.	Agree/ Disagree
13.	I feel enveloped by a dark despair.	Agree/ Disagree
14.	I approach life with hope.	Agree/ Disagree
15.	I feel like I do not see the positive side of many situations.	Agree/ Disagree
16.	Even if I manage to achieve a goal, I cannot enjoy it.	Agree/ Disagree
17.	I feel worthless.	Agree/ Disagree
18.	I often smile honestly and joke.	Agree/ Disagree
19.	I cannot mobilize myself to do anything.	Agree/ Disagree
20.	I feel very insecure.	Agree/ Disagree
21.	I feel that people and the world inspire me.	Agree/ Disagree
22.	It takes some time to me before I realize that something unpleasant is happening around me.	Agree/ Disagree
23.	I feel full of energy.	Agree/ Disagree
24.	I find it difficult to have fun and laugh with others.	Agree/ Disagree
25.	I feel depressed and I cannot escape it.	Agree/ Disagree
26.	I often feel joy.	Agree/ Disagree
27.	I find it difficult to finish things that I have started.	Agree/ Disagree
28.	I feel good with myself.	Agree/ Disagree
29.	I cannot engage in films/novels that cause strong emotions in others.	Agree/ Disagree
30.	I am often so unhappy that it is unbearable.	Agree/ Disagree
31.	I can start new things without difficulty.	Agree/ Disagree
32.	I feel fulfilled.	Agree/ Disagree
33.	Emotional events distract me so much that I later have trouble concentrating.	Agree/ Disagree

34.	I am rarely happy when I get a gift.	Agree/ Disagree
35.	I have plans that I realize willingly.	Agree/ Disagree
36.	I feel emotionally spent.	Agree/ Disagree
37.	I find happiness in small things.	Agree/ Disagree
38.	Everything I do makes me tired.	Agree/ Disagree
39.	I am afraid of many things.	Agree/ Disagree
40.	I am often cheerful.	Agree/ Disagree
41.	I feel like I do not have anything interesting and enjoyable to do.	Agree/ Disagree
42.	I have no appetite.	Agree/ Disagree
43.	I enjoy every day.	Agree/ Disagree
44.	I often feel guilty for various reasons.	Agree/ Disagree
45.	I am full of positive emotions.	Agree/ Disagree
46.	I have great difficulty making decisions.	Agree/ Disagree
47.	I have thought about suicide.	Agree/ Disagree
48.	I feel like I notice nice things in my surroundings more slowly than others, e.g., good weather or beautiful sights.	Agree/ Disagree
49.	I feel lonely and abandoned.	Agree/ Disagree
50.	I have many reasons to be happy.	Agree/ Disagree
51.	I feel like I deserve punishment.	Agree/ Disagree
52.	I feel like the future is hopeless and nothing can change that.	Agree/ Disagree
53.	Many things make me happy.	Agree/ Disagree
54.	I do not see any sense in what I do.	Agree/ Disagree

55.	I feel like I notice ugly things in my surroundings more slowly than others, e.g., cloudy weather or a littered area.	Agree/ Disagree
56.	I feel resentful for many reasons.	Agree/ Disagree
57.	I do not feel like doing anything.	Agree/ Disagree
58.	Contact with people tires me.	Agree/ Disagree
59.	It is easy to make me happy.	Agree/ Disagree
60.	I wish it would all end.	Agree/ Disagree
61.	I can laugh and see the funny side of things.	Agree/ Disagree
62.	Everything seems bland and trivial.	Agree/ Disagree
63.	I am not able to feel happy with things that happen to me.	Agree/ Disagree
64.	I have a zeal for acting.	Agree/ Disagree

Please make sure you did not omit any of the above statements.
