# Supplementary material for: Types of Anxiety and Depression: Theoretical Assumptions and Development of the Anxiety and Depression Questionnaire
Source: Front Psychol. 2018 Jan 23;8:2376. doi: 10.3389/fpsyg.2017.02376 (PMC5787098; doi:10.3389/fpsyg.2017.02376)
Supplement: Supplementary file 2 [file DataSheet2.pdf]

Anxiety and Depression Questionnaire (ADQ-VD)

Małgorzata Fajkowska, Agata Wytykowska, Joanna Kantor-Martynuska
& Ewa Domaradzka

Name and surname/ or nickname ....
Date of birth ....Sex ....
Education ....E-mail address...

This questionnaire contains statements that concern various emotions, experiences, and behaviors. Each statement can be responded to by circling “Agree” or “Disagree”. Please mark the answer that describes you better. Do not spend too much time on individual statements. There are no good or bad responses. Please respond to all of the statements provided below.

1.	I prefer to travel by car rather than by train.	Agree/ Disagree
2.	My impression is that it is sometimes difficult for me to recognize negative emotions in others.	Agree/ Disagree
3.	I am very often tense.	Agree/ Disagree
4.	I often have headaches after social events.	Agree/ Disagree
5.	It is difficult for me to get involved in news and stories that other people find moving.	Agree/ Disagree
6.	I am very often edgy.	Agree/ Disagree
7.	I often feel hurt by what someone says.	Agree/ Disagree
8.	In comparison to others, it is more difficult for me to notice the various nuances in relations between people.	Agree/ Disagree
9.	I often feel overwhelmed by sadness.	Agree/ Disagree
10.	I have the impression that it is sometimes difficult for me to recognize my own negative emotions.	Agree/ Disagree
11.	People often say that I am malicious.	Agree/ Disagree
12.	I feel that I am in a bad mood right now.	Agree/ Disagree

13.	I like to cook.	Agree/ Disagree
14.	When I am among people, I feel like I am ‘‘behind the glass’’.	Agree/ Disagree
15.	Usually when I am talking about something, it lacks emotion.	Agree/ Disagree
16.	I often get angry.	Agree/ Disagree
17.	Sometimes I have trouble telling whether someone has positive or negative attitude towards me.	Agree/ Disagree
18.	I feel that I am afraid of something for no apparent reason.	Agree/ Disagree
19.	It takes some time for me until I realize that a given situation is nice and pleasant.	Agree/ Disagree
20.	Social events tire me.	Agree/ Disagree
21.	I am often anxious and worried.	Agree/ Disagree
22.	I find it difficult to notice that someone is sad.	Agree/ Disagree
23.	I feel a lot of impatience with people.	Agree/ Disagree
24.	I often find out that I did not notice something important about the behavior of another person.	Agree/ Disagree
25.	I feel that it is very easy to hurt me.	Agree/ Disagree
26.	I find it difficult to notice that someone is happy.	Agree/ Disagree
27.	In my relations with people I often feel humiliated.	Agree/ Disagree
28.	Sometimes I have no idea what I am feeling in a given moment.	Agree/ Disagree
29.	I find my relations with others to be ‘’emotionally bland’’.	Agree/ Disagree
30.	It is difficult for me to notice anger in others.	Agree/ Disagree
31.	I do not feel comfortable during social situations, that is why I avoid them.	Agree/ Disagree
32.	I like animals.	Agree/ Disagree
33.	I think that people deliberately do things to me out of spite.	Agree/ Disagree
34.	I often fail to notice that I have hurt someone.	Agree/ Disagree

35.	I am often restless.	Agree/ Disagree
36.	During an argument or conflict, it is difficult for me to recognize what the other side feels.	Agree/ Disagree
37.	I have a favorite film.	Agree/ Disagree
38.	I tend to react with anger towards other people's behaviors.	Agree/ Disagree
39.	I try to avoid group activities.	Agree/ Disagree
40.	When I am watching a film, I cannot distinguish what emotions the main character is experiencing.	Agree/ Disagree

Please make sure you did not omit any of the above statements.
