# Supplementary material for: Types of Anxiety and Depression: Theoretical Assumptions and Development of the Anxiety and Depression Questionnaire
Source: Front Psychol. 2018 Jan 23;8:2376. doi: 10.3389/fpsyg.2017.02376 (PMC5787098; doi:10.3389/fpsyg.2017.02376)
Supplement: Supplementary file 3 [file DataSheet3.pdf]

Anxiety and Depression Questionnaire (ADQ-ArA)

Małgorzata Fajkowska, Agata Wytykowska, Joanna Kantor-Martynuska
& Ewa Domaradzka

Name and surname/ or nickname ....
Date of birth ....Sex ....
Education ....E-mail address..

This questionnaire contains statements that concern various emotions, experiences, and behaviors. Each statement can be responded to by circling "Agree" or "Disagree". Please mark the answer that describes you better. Do not spend too much time on individual statements. There are no good or bad responses. Please respond to all of the statements provided below.

1.	I am rarely tense.	Agree/ Disagree
2.	I do not panic, even in the face of threats and dangers.	Agree/ Disagree
3.	Whenever something scares me, I feel a sudden attack of heat or cold.	Agree/ Disagree
4.	I enjoy reading books.	Agree/ Disagree
5.	I am afraid of new situations more than other people.	Agree/ Disagree
6.	I often get sudden anxiety attacks.	Agree/ Disagree
7.	When I get stressed, I can feel my eyes tear up.	Agree/ Disagree
8.	When I am tense, I do not see what is happening around me.	Agree/ Disagree
9.	I instinctively avoid contact with new people.	Agree/ Disagree
10.	My skin crawls when I hear news about something upsetting.	Agree/ Disagree
11.	I stay calm in difficult situations.	Agree/ Disagree

12.	I sometimes panic.	Agree/ Disagree
13.	I easily focus on negative things.	Agree/ Disagree
14.	When I am scared, I feel tingling and numbness in my body.	Agree/ Disagree
15.	Contact with new people usually makes me uneasy.	Agree/ Disagree
16.	I get anxiety attacks for no reason.	Agree/ Disagree
17.	I have favorite foods.	Agree/ Disagree
18.	When something unexpected scares me, I feel like my legs collapse underneath me.	Agree/ Disagree
19.	When I am afraid of something, it is usually difficult for me to breathe deeply.	Agree/ Disagree
20.	I am terribly afraid of heights.	Agree/ Disagree
21.	I prefer the sea to the mountains.	Agree/ Disagree
22.	When I am scared, I feel pain in my chest.	Agree/ Disagree
23.	When I get stressed, I cannot swallow anything.	Agree/ Disagree
24.	When someone unexpectedly asks me something, I do not know what to say, even if I know the answer.	Agree/ Disagree
25.	Things that I am afraid of immediately draw my attention.	Agree/ Disagree
26.	It is difficult to scare me.	Agree/ Disagree
27.	When I am afraid of something, I feel dizzy.	Agree/ Disagree
28.	When I am stressed, I feel like I am choking.	Agree/ Disagree
29.	I do not get anxiety or panic attacks.	Agree/ Disagree
30.	When I notice a potential threat, I automatically withdraw from the given situation.	Agree/ Disagree
31.	My hands and legs shake from fear.	Agree/ Disagree
32.	When I fear something, I often feel paralyzed.	Agree/ Disagree
33.	When I am afraid, I feel like I am about to die.	Agree/ Disagree

34.	I like learning foreign languages.	Agree/ Disagree
35.	When somebody says something to me unexpectedly, I usually shudder.	Agree/ Disagree
36.	When I get scared, I become drenched in sweat.	Agree/ Disagree
37.	When I know that others are watching, doing things is difficult for me.	Agree/ Disagree
38.	When I am tense, I often do not notice signs of danger.	Agree/ Disagree
39.	When I accidentally meet someone that I am afraid of, I feel like my legs give out.	Agree/ Disagree
40.	Anxiety keeps me from speaking without preparation.	Agree/ Disagree
41.	Stress makes my stomach turn.	Agree/ Disagree
42.	I jitter when I am stressed by something.	Agree/ Disagree
43.	My voice trembles when I am stressed.	Agree/ Disagree
44.	I often panic.	Agree/ Disagree
45.	When something unsettling is going on, I can immediately feel it in my body.	Agree/ Disagree

Please make sure you did not omit any of the above statements.
