# Supplementary material for: Types of Anxiety and Depression: Theoretical Assumptions and Development of the Anxiety and Depression Questionnaire
Source: Front Psychol. 2018 Jan 23;8:2376. doi: 10.3389/fpsyg.2017.02376 (PMC5787098; doi:10.3389/fpsyg.2017.02376)
Supplement: Supplementary file 4 [file DataSheet4.pdf]

Anxiety and Depression Questionnaire (ADQ-ApA)

Małgorzata Fajkowska, Agata Wytykowska, Joanna Kantor-Martynuska
& Ewa Domaradzka

Name and surname/ or nickname ....
Date of birth ....Sex ....
Education ....E-mail address..

This questionnaire contains statements that concern various emotions, experiences, and behaviors. Each statement can be responded to by circling “Agree” or “Disagree”. Please mark the answer that describes you better. Do not spend too much time on individual statements. There are no good or bad responses. Please respond to all of the statements provided below.

1.	When trying to concentrate on something, it is difficult for me to get rid of distracting thoughts.	Agree/ Disagree
2.	When I start to worry, I cannot stop.	Agree/ Disagree
3.	During a stressful and nervous situation, I feel tension in my body.	Agree/ Disagree
4.	I find it difficult to forget unpleasant events.	Agree/ Disagree
5.	I cannot concentrate on a difficult task if there are noises around.	Agree/ Disagree
6.	I don’t worry in advance.	Agree/ Disagree
7.	Stress causes me diverse physical ailments.	Agree/ Disagree
8.	When I am under stress, I cannot gather my thoughts.	Agree/ Disagree
9.	Many situations that involved me cause me to worry.	Agree/ Disagree
10.	When facing danger, I often feel like my legs ‘’turn to jelly’’.	Agree/ Disagree
11.	I find it easy to forget about unpleasant events.	Agree/ Disagree
12.	When I am excited or upset because of something I find it difficult to turn my attention away.	Agree/ Disagree
13.	My body reacts intensively to sudden stress.	Agree/ Disagree

14.	I know I should not worry about certain things, but I cannot help it.	Agree/ Disagree
15.	It is difficult for me to drop my current way of thinking and look at a problem from a different point of view.	Agree/ Disagree
16.	I worry a lot when I am under pressure.	Agree/ Disagree
17.	I often rethink my most recent problems.	Agree/ Disagree
18.	My concerns overwhelm me.	Agree/ Disagree
19.	When something unpleasant happens to me I find it difficult to relax, even among other people.	Agree/ Disagree
20.	My hands are cold or sweaty when I am stressed.	Agree/ Disagree
21.	It is difficult for me to focus on a current task.	Agree/ Disagree
22.	Often a persistent thought is tormenting me.	Agree/ Disagree
23.	As soon as I am done with something I start worrying about what I have left to do.	Agree/ Disagree
24.	I analyze various unpleasant situations in detail.	Agree/ Disagree
25.	It is easy to turn my attention away from what I am doing.	Agree/ Disagree
26.	I constantly worry about something.	Agree/ Disagree
27.	I forget about my failures quickly.	Agree/ Disagree
28.	When I am worrying about something I have difficulty concentrating on work.	Agree/ Disagree
29.	I quickly lose my cool and temper in difficult situations.	Agree/ Disagree
30.	An unpleasant situation can keep me from work for a few hours.	Agree/ Disagree
31.	I find it difficult to accept failure.	Agree/ Disagree
32.	I worry about what others might say about me.	Agree/ Disagree
33.	When I get stressed, I cannot speak in a calm voice.	Agree/ Disagree
34.	When I have a family or professional problem, I think about it constantly.	Agree/ Disagree
35.	I am often tense.	Agree/ Disagree

36.	I can easily push away negative thoughts.	Agree/ Disagree
37.	I think that I deal with most things worse than others.	Agree/ Disagree
38.	When a distracting thought comes to my mind I can easily turn my attention away from it.	Agree/ Disagree
39.	It is easy to scare me.	Agree/ Disagree
40.	I usually stop thinking about negative things quickly.	Agree/ Disagree
41.	My friends tell me I worry too much about everything.	Agree/ Disagree
42.	I often imagine the worst case scenario.	Agree/ Disagree
43.	When something unsettling is happening, I immediately feel it in my body.	Agree/ Disagree
44.	I think that I have more worries than others.	Agree/ Disagree
45.	When I fail at something, I cannot function normally for several days.	Agree/ Disagree
46.	Sudden stress paralyzes me.	Agree/ Disagree
47.	I am not in the habit of worrying excessively.	Agree/ Disagree
48.	When something unpleasant happens, I cannot stop thinking about it.	Agree/ Disagree

Please make sure you did not omit any of the above statements.
